# Accurate Diabetes Risk Stratification Using Machine Learning: Role of Missing Value and Outliers

**DOI:** 10.1007/s10916-018-0940-7

**Published:** 2018-04-10

**Authors:** Md. Maniruzzaman, Md. Jahanur Rahman, Md. Al-MehediHasan, Harman S. Suri, Md. Menhazul Abedin, Ayman El-Baz, Jasjit S. Suri

**Affiliations:** 1 Department of Statistics, University of Rajshahi, Rajshahi, Bangladesh; 2 The JiVitA Project of Johns Hopkins University, Gaibandha, Bangladesh; 3 Department of Computer Science and Engineering, Rajshahi University of Engineering and Technology, Rajshahi, Bangladesh; 4 Brown University, Providence, RI USA; 5 Statistics Discipline, Khulna University, Khulna, Bangladesh; 6 Department of Bioengineering, University of Louisville, Louisville, KY USA; 7 Stroke Monitoring and Diagnostic Division, AtheroPoint LLC, Roseville, CA USA; 8 Knowledge Engineering Center, Global Biomedical Technologies, Roseville, CA USA

**Keywords:** Diabetes, Missing values, Outliers, Risk stratification, Feature selection, Machine learning

## Abstract

Diabetes mellitus is a group of metabolic diseases in which blood sugar levels are too high. About 8.8% of the world was diabetic in 2017. It is projected that this will reach nearly 10% by 2045. The major challenge is that when machine learning-based classifiers are applied to such data sets for risk stratification, leads to lower performance. Thus, our objective is to develop an optimized and robust machine learning (ML) system under the assumption that missing values or outliers if replaced by a median configuration will yield higher risk stratification accuracy. This ML-based risk stratification is designed, optimized and evaluated, where: (i) the features are extracted and optimized from the six feature selection techniques (random forest, logistic regression, mutual information, principal component analysis, analysis of variance, and Fisher discriminant ratio) and combined with ten different types of classifiers (linear discriminant analysis, quadratic discriminant analysis, naïve Bayes, Gaussian process classification, support vector machine, artificial neural network, Adaboost, logistic regression, decision tree, and random forest) under the hypothesis that both missing values and outliers when replaced by computed medians will improve the risk stratification accuracy. Pima Indian diabetic dataset (768 patients: 268 diabetic and 500 controls) was used. Our results demonstrate that on replacing the missing values and outliers by group median and median values, respectively and further using the combination of random forest feature selection and random forest classification technique yields an accuracy, sensitivity, specificity, positive predictive value, negative predictive value and area under the curve as: **92.26%, 95.96%, 79.72%, 91.14%, 91.20%,** and **0.93**, respectively. This is an improvement of 10% over previously developed techniques published in literature. The system was validated for its stability and reliability. RF-based model showed the best performance when outliers are replaced by median values.

**Electronic supplementary material:**

The online version of this article (10.1007/s10916-018-0940-7) contains supplementary material, which is available to authorized users.

## Introduction

Diabetes mellitus (DM) is known as diabetes in which blood glucose levels are too high [1]. As a result, the disease increases the risk of cardiovascular diseases such as heart attack and stroke etc. [2]. There were about 1.5 million deaths directly due to diabetes and 2.2 million deaths due to cardiovascular diseases, chronic kidney disease, and tuberculosis in 2012 [3]. Unfortunately, the disease is never cured but can be managed by controlling glucose. About 8.8% of adults worldwide were diabetic in 2017 and this number is projected to be 9.9% in 2045 [4]. There are three kinds of diabetes disease: (i) juvenile diabetes (type I diabetes), (ii) type II diabetes, and (iii) type III diabetes (gestational diabetes) [5]. In type I diabetes, the body does not produce proper insulin. Usually, it is diagnosed in children and young adults [6]. Type II diabetes usually develops in adults over 45 years, but also in young age children, adolescents and young adults. With type II diabetes, the pancreas does not produce enough insulin. Almost 90% of all diabetes is type II [7]. The third type of diabetes is gestational diabetes. Pregnant women, who never had diabetes before, but have high blood glucose levels during pregnancy are diagnosed with gestational diabetes.

Diabetic classification is an important and challenging issue for the diagnosis and the interpretation of diabetic data [8]. This is because the medical data is nonlinear, non-normal, correlation structured, and complex in nature [9]. Further, the data has missing values or has outliers, which further affects the performance of machine learning systems for risk stratification. A variety of different machine learning techniques have been developed for the prediction and diagnosis of diabetes disease such as: linear discriminant analysis (LDA), quadratic discriminant analysis (QDA), naïve Bayes (NB), support vector machine (SVM), artificial neural network (ANN), feed-forward neural network (FFNN), decision tree (DT), J48, random forest (RF), Gaussian process classification (GPC), logistic regression (LR), and k-nearest neighborhood (KNN) [9, 10]. These classifiers cannot correctly classify diabetic patients when the data contains missing values or has outliers, and therefore, when the machine learning-based classifiers are used for risk stratification, it does not yield higher accuracy [10–16].

In statistics, outlier removal and the handling of missing values is an important issue and have never been ignored. Previous machine learning techniques [10] have been unsuccessful mainly because their classifications are either (a) directly on the raw data without feature extraction or (b) on raw data without outlier removal or (c) without adding replacement values for missing values or (d) filling missing values simply with the mean value. Moreover, outlier replacements using computed mean is very sensitive [11]. As a result, their classification accuracy is low. Several authors tried outlier removal or the filling of missing values, but in the non-classification framework [12–16]. Our techniques were motivated by the spirit of these statistical measures embedded in a classification framework. To improve the classification accuracy, we adapted a missing value approach based on group median, outlier removal using medians, and further optimizing the data set by choosing the combination of best feature selection criteria and classification model among the set of six feature selection techniques and ten classification models.

**Fig. 1:**
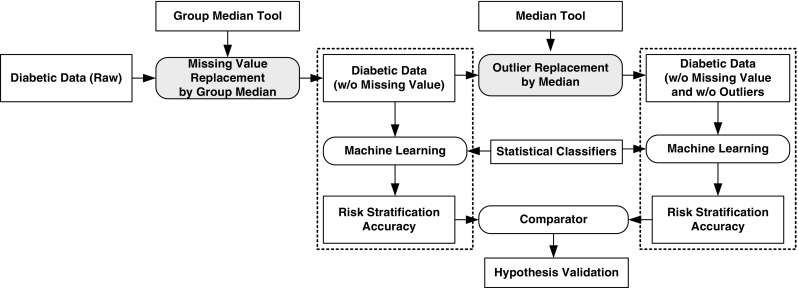
Preparation of diabetic data by missing value replacement and outlier removal

The hypothesis has been laid out in Fig. 1, where input diabetic data undergoes two stage process of data preparation: (i) missing value process to replace the missing value by the group median and (ii) removal of the outliers by the median values. The filtered data then undergoes machine learning risk stratification paradigm, given the set of classifiers. The comparator helps in comparing the classification accuracy when the data has (a) no missing values but has outliers against classification accuracy when the data (b) has no missing values and no outliers.

Among the set of classifiers, we adapted RF [17] to extract and select significant features and also predict diabetic disease using the RF-based classifier. RF-based classifier is the most powerful machine learning technique in both classification and regression [18]. Some key strengths of RF are: (i) suites nonlinear and non-normal data; (ii) avoids over fitting of the data; (iii) provides robustness to noise; (iv) possesses an internal mechanism to estimate error rates; (v) provides the rank of variable importance; (vi) adaptable on both continuous and categorical variables; and (vii) fits well for data imputation and cluster analysis. In our current study, we hypothesize that by (a) replacing missing values with group median and outliers by median, and (b) using feature extraction by RF combined with the RF-based classifier will lead to the highest accuracy and sensitivity compared to conventional techniques like: LDA, QDA, NB, GPC, SVM, ANN, Adaboost, LR, and DT. The performances of these classifiers have been evaluated by using accuracy (ACC), sensitivity (SE), specificity (SP), positive predictive value (PPV), negative predictive value (NPV) and area under the curve (AUC).

Thus, following are the novelties of our current study compared to the previous studies:

1. Design of ML system where, one can remove missing values using group median, check outliers by using inter-quartile range (IQR) and if there exit outliers, replace outliers with the median values.
2. Optimizing the ML system by selecting the best combination of feature selection and classification model among the six features selection techniques (random forest (RF), logistic regression (LR), mutual information (MI), principal component analysis (PCA), analysis of variance (ANOVA), and Fisher discriminant ratio (FDR)) and ten classification models (RF, LDA, QDA, NB, GPC, SVM, ANN, AB, LR, and DT).
3. Understanding the different cross-validation protocols (K2, K4, K5, K10, and JK) for determining the generalization of the ML system and computing the performance parameters such as: ACC, SE, SP, PPV, NPV, and AUC.
4. Demonstration of automated reliability index (RI) and stability index, which are used to check the validity of our study and further, benchmarking our ML system against the existing literature.
5. Demonstration of an improvement in classification accuracy compared against current techniques available in literature by **10%** using K10 protocol and **18%** using JK protocol under the combination of current framework.

The overall layout of this paper is as follows: Section 2 represents the patient’s demographics, section 3 represents methodology, including feature selection methods and classification methods are discussed in this section. Experimental protocols are given in section 4. Results are discussed in section 5. Section 6 represents the hypothesis validations and performance evaluation. Section 7 represents the discussions in detail and finally conclusion is presented in section 8.

## Patient demographics

The diabetic dataset has been taken from the University of California, Irvine (UCI) Repository. This dataset consists of 768 female patients, at least 21 years old of Pima Indian heritage, having 268 diabetic patients and 500 controls. In this dataset, five patients have zero glucose level, diastolic blood pressure is zero in 35 patients, 27 patients have zero body mass indexes, 227 patients have zero skin fold thickness and 374 patients have zero serum insulin level. These zero values have no meaning and is treated as missing values. As a preprocessing step, we divide the dataset into two parts: diabetic and control, and then the missing values are replaced by the median of each group. We also check the outliers by inter-quartile range (IQR). If outliers exist, we have replaced outliers by the median. The flow chart of data preparations is described in Fig. 1. The descriptions of the attributes and brief statistical summary are shown in Table 1.

**Table 1:** Demographics of the diabetic patient cohort

SN	Attributes	Descriptions	Attributes type’s	Mean ± SD
1	Pregnant	Number of times pregnant	Continuous	3.84 ± 3.36
2	Glucose	Plasma glucose (2-h)	Continuous	121.67 ± 30.46
3	Pressure	Diastolic blood pressure (mm Hg)	Continuous	72.38 ± 12.10
4	Triceps	Triceps skin fold thickness (mm)	Continuous	29.08 ± 8.89
5	Insulin	Two hours serum-insulin (μ U/ml)	Continuous	141.76 ± 89.10
6	Mass	Body mass index (weight in kg/ (height in m)^2^)	Continuous	32.43 ± 6.88
7	Pedigree	Diabetes pedigree function	Continuous	0.47 ± 0.33
8	Age	Age (years)	Continuous	33.24 ± 11.76
9	Class	Diabetic (500) vs. control (268)	Categorical	–

## Methodology

**Fig. 2:**
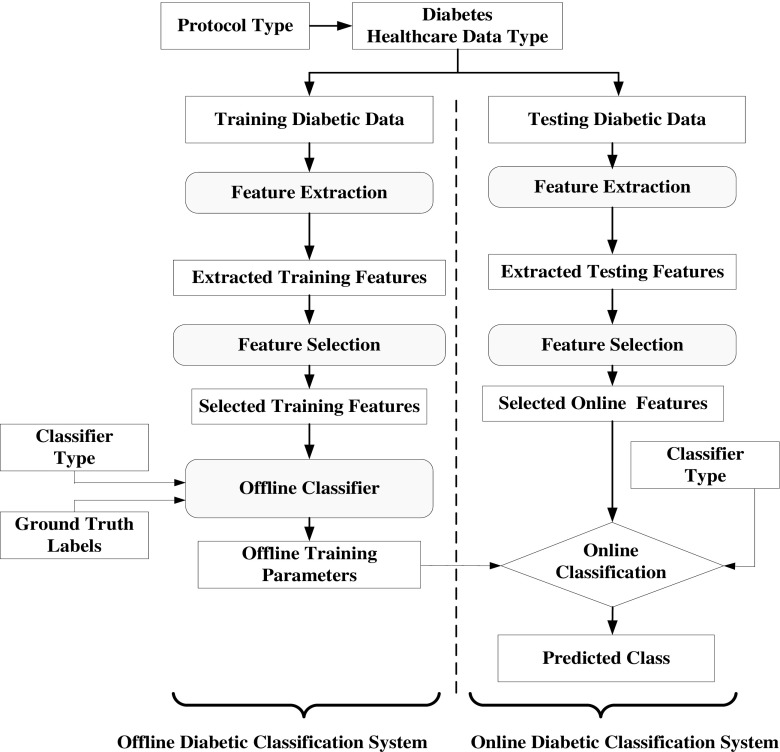
Architecture of the machine learning system

The idea of proposed overall machine learning system is presented in Fig. 2. This follows the conventional model of ML; however the input data is now preprocessed by taking care of missing values and outlier removal. The dotted line divides the system into two segments: training diabetic data or offline (shown on the left) and testing diabetic data or online system (shown on right). The basic difference between the training and testing protocol is that the training system works on the basis of a priori ground truth and testing protocols perform prediction of diabetes. The next stage is the feature extraction followed by feature selection block, whose role is to diminish the system complexity while choosing the dominant features. Six types of feature selection techniques have been adapted, i.e., RF, LR, MI, PCA, ANOVA, and FDR. The features are trained based on the binary class framework model. Using the training database and ground truth, the machine learning parameters use online classifiers (classifier types) such as: LDA, QDA, NB, GPC, SVM, ANN, Adaboost, LR, DT, and RF. These training-based machine learning parameters and dominant features extracted from the test datasets are transformed to predict of diabetic patients.

### Feature selection methods

Feature selection is important in the field of machine learning. Often in data science, we have hundreds or even millions of features and we want a way to create a model that only includes the most informative features. It has three benefits as (i) we easily run our model to interpret; (ii) reduce the variation of the model; and (iii) reduce the computational cost and time of the training model. The optimal feature selection removes the complexity of the system and increases the reliability, stability, and classification accuracy. The main feature selections methods are used: PCA, ANOVA, FDR, MI, LR, and RF, presented below:

#### Principal component analysis

Feature selection technique (FST) always removes the less dominant features and improves the classification accuracy and reduces the computational cost and time consumption of machine learning algorithm. Principal component analysis (PCA) is one of the popular dimension reduction technique. In this study, we adapted pooling methodology along with PCA [19] which extract the important features. The PCA algorithm of feature selection is given below:

1. Calculate the mean vectors across each feature space dimension as:

1 $$ {\boldsymbol{\upmu}}_{\left(\mathrm{P}\times 1\right)}=\frac{1}{\mathrm{N}}{\mathbf{X}}^{\mathbf{T}}\mathbf{I} $$

Here, **X** is a matrix of N × P, where, N is a total number of patients, P is the total number of attributes, and **I** is a vector of 1’s of size N × 1.

2. To make normalize the data (i.e., zero mean and unit variance), we subtract mean vectors from data matrix as:

2 $$ {\mathbf{A}}_{\left(\mathrm{N}\times \mathrm{P}\right)}=\mathbf{X}-\boldsymbol{\upmu} $$

3. Compute the covariance matrix of the dataset by using formula

3 $$ {\mathbf{S}}_{\left(\mathrm{P}\times \mathrm{P}\right)}=\frac{1}{\mathrm{N}}{\mathbf{A}}^{\mathbf{T}}\mathbf{A} $$

4. Compute the eigenvalues (λ_1_, λ_2_, …, λ_P_) and eigenvectors (e_1_, e_2_, …, e_P_) of the covariance matrix (**S**).
5. Sort the eigenvalues in descending order and arrange the corresponding eigenvectors in the same order.
6. Choose the number of principal components (m) to be considered using the following criterion:

4 $$ \frac{\sum_{\mathrm{i}=1}^{\mathrm{m}}{\uplambda}_{\mathrm{i}}}{\sum_{\mathrm{i}=1}^{\mathrm{P}}\uplambda \mathrm{i}}>\mathrm{R} $$

where, R is the cutoff point varying from 0.90 to 0.95, P is the total number of eigenvalues.

7. Compute the contribution of each feature as the following dominance indices:

5 $$ {\mathrm{b}}_{\mathrm{n}}=\sum \limits_{\mathrm{z}=1}^{\mathrm{m}}\left|{\mathrm{e}}_{\mathrm{z}\mathrm{n}}\right| $$

where, e_zn_ indicates the n^th^ entry of e_n_ which is the z^th^ eigenvectors, *n* = 1, 2… P and |e_zn_|  shows the absolute value of e_zn_.

Sort the indices b_n_ in descending order and select first m features which will give the reduced number of features (m) (without modifying original feature values) with their dominance level from highest to lowest.

#### Analysis of variance

The main goal of one-way analysis of variance (ANOVA) test is to perform tests whether or not all the different classes of **Y** have the same mean as **X**. To perform ANOVA-test, the following notations are used.

N_j_: Number of classes with **Y** = j.
**μ**_j_: The sample mean of the predictors **X** for the target variables **Y** = j.
$$ {\mathbf{S}}_{\mathrm{j}}^2 $$
The sample variance of the predictors **X** for the target variables **Y** = j:

6 $$ {\mathbf{S}}_{\mathrm{j}}^2=\frac{\sum_{\mathrm{j}=}^{{\mathrm{N}}_{\mathrm{j}}}{\left({\mathbf{X}}_{\mathrm{ij}}-{\boldsymbol{\upmu}}_{\mathrm{j}}\right)}^2}{{\mathrm{N}}_{\mathrm{j}}-1} $$

**μ**= The overall mean of the predictors **X**: $\boldsymbol{\upmu} =\frac{\sum_{\mathrm{j}=1}^{\mathrm{N}}{\mathrm{N}}_{\mathrm{j}}{\mathrm{X}}_{\mathrm{j}}}{\mathrm{N}}$, where N is the total number of patients and J are the total number of classes. The *p*-value is calculated based on the F-statistic which *p*-value is = Prob.{F (J-1, N-1) > F} where, $\mathrm{F}=\frac{\frac{\sum_{\mathrm{j}=1}^{\mathrm{J}}{\mathrm{N}}_{\mathrm{j}}{\left({\boldsymbol{\upmu}}_{\mathrm{j}}-\boldsymbol{\upmu} \right)}^2}{\left(\mathrm{J}-1\right)}}{\frac{\sum_{\mathrm{j}=1}^{\mathrm{J}}\left({\mathrm{N}}_{\mathrm{j}}-1\right){\mathrm{S}}_{\mathrm{j}}^2}{\left(\mathrm{N}-1\right)}}$ which follows F-distribution with (J-1) and (N-1) degrees of freedom respectively. We select the features whose *p*-values are less than 0.0001.

#### Fisher discriminant ratio

Fisher discriminant ratio (FDR) selects the most informative features in such a way that the distance between the data points of within-class should be as large as possible, while the distance between the data points between-class should be as small as possible [20]. The general algorithm of FDR in details is given below.

1. Calculate the sample mean vectors **μ**_j_ of the different class:

7 $$ {\boldsymbol{\upmu}}_{\mathrm{j}}=\frac{1}{{\mathrm{N}}_{\mathrm{j}}}{\sum \limits}_{\mathrm{X}\in {\mathrm{D}}_{\mathrm{j}}}^{\mathrm{N}}{\mathbf{X}}_{\mathrm{k}}\kern1em ;\mathrm{j}=1,2. $$

2. Compute the scatter matrices (in-between-class and within-class scatter matrix). The within-class scatter matrix **S**_w_ is calculated by the following formula:

8 $$ {\mathbf{S}}_{\mathrm{w}}=\sum \limits_{\mathrm{j}=1}^{\mathrm{K}}{\mathbf{S}}_{\mathrm{j}},\kern0.75em \mathrm{where},{\mathbf{S}}_{\mathrm{j}}=\sum \limits_{\mathrm{X}\in {\mathrm{D}}_{\mathrm{j}}}^{\mathrm{N}}\left(\mathbf{X}-{\boldsymbol{\upmu}}_{\mathrm{j}}\right){\left(\mathbf{X}-{\boldsymbol{\upmu}}_{\mathrm{j}}\right)}^{\mathrm{T}} $$

3. The between-class scatter matrix **S**_B_ is computed by the following:

9 $$ {\mathbf{S}}_{\mathrm{B}}=\sum \limits_{\mathrm{j}=1}^{\mathrm{K}}{\mathrm{N}}_{\mathrm{j}}\left({\boldsymbol{\upmu}}_{\mathrm{j}}-\boldsymbol{\upmu} \right){\left({\boldsymbol{\upmu}}_{\mathrm{j}}-\boldsymbol{\upmu} \right)}^{\mathrm{T}} $$

where, **μ** is the overall mean vectors, **μ**_j_ is the j^th^ sample mean vectors and N_j_ is the number of classes the respective patients.

4. Finally, the FDR is computed by comparing the relationship between the within-class scatter and between-class scatter matrix by the following formula:

$$ \mathrm{FDR}={\mathbf{S}}_{\mathrm{W}}^{-1}{\mathbf{S}}_{\mathrm{B}} $$

5. Compute the eigenvalues (λ_1_, λ_2_, …, λ_P_) and the corresponding eigenvectors (e_1_, e_2_, …, e_P_) for the scatter matrices (FDR=${\mathbf{S}}_{\mathrm{W}}^{-1}{\mathbf{S}}_{\mathrm{B}}$**)**.
6. Sort the eigenvectors by decreasing eigenvalues and choose number of K eigenvectors with the largest eigenvalues to form a P× K dimensional weighted matrix **W** (where every column represents an eigenvector).
  Use this P× K eigenvector matrix to transform the samples into the new subspace. This can be summarized as follows:

10 $$ \mathbf{Y}=\mathbf{XW} $$

where, **X** is a N× P-dimensional matrix representing the N samples, and **Y** is the N× K-dimensional samples in the new spaces.

#### Mutual information

Mutual information (MI) is a well-known dependence measure in information theory. It detects a subset of most informative features [21]. It requires two parameters as its input i.e.*,* the numbers of most informative features to be selected for classification and the number of quantization levels into which the continuous features are binned. Due to redundancy in features, there is over-fitting, and therefore dominant features are selected via this technique. In our current study, the numbers of the important features are selected for our classifier by using t-test based on *p*-values which are less than 0.0001. For two discrete variables x and y, the mutual information is denoted my MI (x, y) and is defined as:

11 $$ \mathrm{MI}\ \left(\mathrm{x},\mathrm{y}\right)=\sum \limits_{\mathrm{i},\mathrm{j}}\mathrm{p}\left({\mathrm{x}}_{\mathrm{i}},{\mathrm{y}}_{\mathrm{j}}\right)\log \frac{\mathrm{p}\left({\mathrm{x}}_{\mathrm{i}},{\mathrm{y}}_{\mathrm{j}}\right)}{\mathrm{p}\left({\mathrm{x}}_{\mathrm{i}}\right)\mathrm{p}\left({\mathrm{y}}_{\mathrm{j}}\right)} $$

where, p(x, y) is the joint probability distributions of x and y, p(x) and p(y) are the marginal probability distribution of x and y.

#### Logistic regression

Logistic regression (LR) is used when the dependent variable is categorical. The logistic model is used to estimate the probability of a binary response based on one or more predictor variables. We estimate the coefficients of the logistic regression by applying maximum likelihood estimator (MLE) and test the coefficients by applying the z-test. We select the features corresponding to the coefficients where *p*-values are less than 0.0001.

#### Random forest

Random forest (RF) directly performs feature selection while the classification rules are built. There are two methods used for variable importance measurements as (i) Gini importance index (GIM), and (ii) permutation importance index (PIM) [22]. In this study, we have used two steps to select the important features: (i) PIM index is used to order the features and (ii) RF is used to select the best combination of features for classification [17]. These same techniques are used on both types of data: data with outlier O1 and data without outlier O2. These reduced features are used for classification.

### Ten classification models

**Fig. 3:**
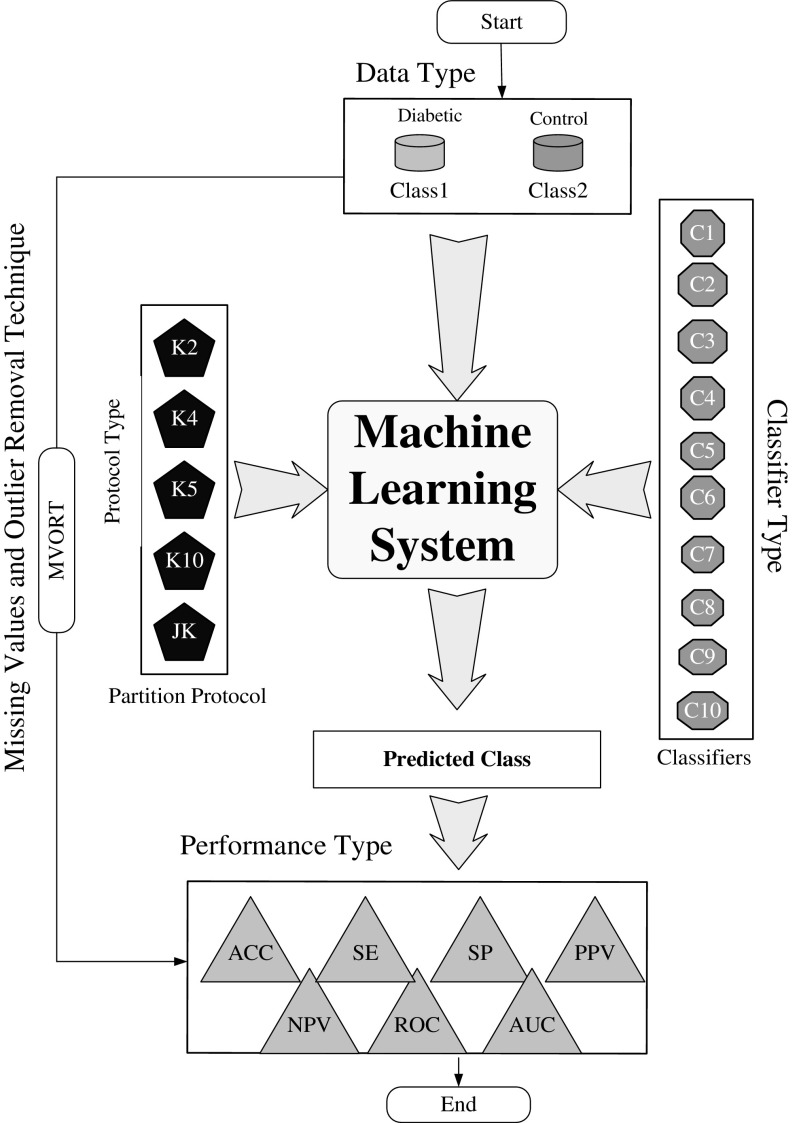
Concept showing the hypothesis link between outlier removals in relation to the performance of the ML system

Ten classification techniques have been adapted for risk stratification in machine learning framework. They are adapted as per their simplicity and popularity: LDA, QDA, NB, GPC, SVM, ANN, Adaboost, LR, DT, and RF. We also adapted five sets of cross-validation protocols as K2, K4, K5, K10, and JK, respectively, and repeated these protocol 10 trials (T). These above systems are implemented under two different sets of paradigms: while outliers (O1) are present and impute outliers by median (O2). Monitoring outputs of the performance system yields ACC, SE, SP, PPV, NPV, and AUC of ROC which is shown in Fig. 3. Brief discussions on the classifiers are presented here:

#### Classifier type 1: Linear discriminant analysis

Ronald Aymer Fisher introduced the linear discriminant analysis (LDA) in 1936. It is an effective classification technique. It classifies n-dimensional space into two-dimensional space that is separated by a hyper-plane. The main objective of this classifier is to find the mean function for every class. This function is projected on the vectors that maximizes the between-groups variance and minimizes the within-group variance [23].

#### Classifier type 2: Quadratic discriminant analysis

Quadratic discriminant analysis (QDA) is used in machine learning and statistical learning to classify two or more classes by a quadric surface. It is distance based classification techniques and it is an extension of LDA. Unlike LDA, there is no assumption that the covariance matrix for every class is identical. When the normality assumption is true, the best possible test for the hypothesis that a given measurement is from a given class is the likelihood ratio test [24].

#### Classifier type 3: Naïve bayes

Naïve Bayes (NB) classifier is a powerful and straightforward classifier and particularly useful in large-scale dataset. It is used on both machine learning and medical science (especially, diagnosis of diabetes). It is a probabilistic classifier based on Bayes’ theorem with the strong independent assumption between the features. It is assumed that the presence of particular features in a class is unrelated to any other features [25].

#### Classifier type 4: Gaussian process classification

In the last decade, Gaussian process (GP) has become a powerful, nonparametric tool that is not only used in regression but also in classification problems in order to handle various problems such as insufficient capacity of the classical linear method, complex data types, the curse of dimension, etc. The main advantages of this method are the ability to provide uncertainty estimates and to learn the noise and smoothness parameters from training data. A GP-based supervised learning technique attempts to take benefit of the better of two different schools of techniques: SVM developed by Vapnik in the early nineties of the last century and Bayesian methods. A GP is a collection of random variables, any finite number of which has a joint Gaussian distribution. A GP is a Gaussian random function and is fully specified by a mean function and covariance function [26]. In our current study, we have used the radial basis kernel (RBF).

#### Classifier type 5: Support vector machine

Support vector machine (SVM) is a supervised learning technique and widely used in medical diagnosis for classification and regression [27]. SVM minimizes the empirical classification error and maximizes the margin, called hyper-plane between two parallel hyper-planes. The classification of a non-linear data is performed using the kernel trick that maps the input features into high-dimensional space. In our current study, we have used the radial basis kernel (RBF).

#### Classifier type 6: Artificial neural network

The concept of the artificial neural network (ANN) [28] is inspired by the biological nervous system. The ANN has following key advantage: (i) it is a data driven, self-adaptive method, i.e., it can adjust themselves to the data and (ii) it is a non-linear model, which makes it flexible in modeling real-world problem. In our current study, we have used back propagation algorithm for training ANN and 10 hidden layers to find better results.

#### Classifier type 7: Adaboost

Adaboost means adaptive boosting, is a machine learning technique. Yoav Freund and Robert Schapire formulated Adaboost algorithm and won golden prizes in 2003 for their work. It can be used in conjunction with different types of algorithm to improve classifier’s performance. Adaboost is very sensitive to handle noisy data and outliers. In some problems, it can be less susceptible to the over fitting problem than other learning algorithms. Every learning algorithm tends to suit some problem types better than others, and typically has many different parameters and configurations to adjust before it achieves optimal performance on a dataset. Adaboost is known as the best out-of-the-box classifier [29].

#### Classifier type 8: Logistic regression

Logistic regression (LR) is basically a linear model for classification rather than regression. It is a basic model which describes dummy output variables and can be extended for diabetes disease classification [30]. The main advantages of LR are that it is more robust and it may handle non-linear data. Let us consider there are N input features like X_1_, X_2_…, X_N_, and P is the probability of the event that will occur and 1-P is the probability of the event that is not occurred. The mathematical expression of the model as follows:

12 $$ \log \left(\frac{\mathrm{P}}{1-\mathrm{P}}\right)=\mathrm{logit}\ \left(\mathrm{P}\right)={\upbeta}_0+{\upbeta}_1{\mathrm{X}}_1+,\dots \dots, +{\upbeta}_{\mathrm{N}}{\mathrm{X}}_{\mathrm{N}} $$

where, **β**_0_ is the intercept term and **β**_i_ (*i* = 1, 2, 3,…, N) is the regression coefficients.

#### Classifier type 9: Decision tree

A decision tree (DT) classifier is a decision support tool that uses a tree structure this is built using input features. The main objective of this classifier is to build a model that predicts the target variables based on several input features. One can easily extract decision rules for a given input data which makes this classifier suitable for any kinds of application [31].

#### Classifier type 10: Random forest

Random forest (RF) is one of the popular supervised techniques in the field of machine learning. It is also an ensemble a multitude of decision trees at training time that outputs the class that is the mode of the classes for classification or average mean prediction for regression of the individual trees [18]. The algorithm of RF is given as follows.

Step 1: For a given training dataset, extract a new sample set by repeated N time’s using bootstrap method. For example, we sample of (X_1_, Y_1_),…, (X_N_, Y_N_) from a given training dataset (X_1_, Y_1_),…,(X_n_, Y_n_). Samples are not extracted consisting of out of bag data (OOB).
Step 2: Build a decision tree based on the results of step 1.
Step 3: Repeat step 1 and step 2 and results in many trees (here 100 trees used) and comprise a forest.
Step 4: Let every tree in the forest to vote for X_i_.
Step 5: Calculate the average of votes for every class and the class with the highest number of votes is the classification label for X.
Step 6: The percentage of correct classification is the accuracy of RF.

### Statistical evaluation

Performances of all classifiers are evaluated by different measurement factors as accuracy (ACC), sensitivity (SE), specificity (SP), positive predictive value (PPV), negative predictive value (NPV) etc. These measurement factors are calculated by using true positive (TP), true negative (TN), false positive (FP), and false negative (FN). Using these measures, the performance measures can be defined as

- *Accuracy*

It is the proportion of the sum of the true positive and true negative against total number of population. It can be expressed mathematically as follows:

13 $$ \mathrm{ACC}\ \left(\%\right)=\left(\frac{\mathrm{TP}+\mathrm{TN}}{\mathrm{TP}+\mathrm{FN}+\mathrm{FP}+\mathrm{TN}}\right)\times 100 $$

- *Sensitivity*

It is the proportion of the positive condition against the predicted condition is positive. It can be expressed mathematically as follows:

14 $$ \mathrm{SE}\ \left(\%\right)=\left(\frac{\mathrm{TP}}{\mathrm{TP}+\mathrm{FN}}\right)\times 100 $$

- *Specificity*

It is the proportion of the negative condition against the predicted condition is negative. It can be expressed mathematically as follows:

15 $$ \mathrm{SP}\ \left(\%\right)=\left(\frac{\mathrm{FP}}{\mathrm{FP}+\mathrm{TN}}\right)\times 100 $$

- *Positive predictive value*

The positive predictive value is the proportion of the predicted positive condition against the true condition is positive. It can be expressed mathematically as follows:

16 $$ \mathrm{PPV}\ \left(\%\right)=\left(\frac{\mathrm{TP}}{\mathrm{TP}+\mathrm{FP}}\right)\times 100 $$

- *Negative predictive value*

It is the proportion of the predicted negative condition against the true condition is negative. It can be expressed mathematically as follows:

17 $$ \mathrm{NPV}\ \left(\%\right)=\left(\frac{\mathrm{TN}}{\mathrm{FN}+\mathrm{TN}}\right)\times 100 $$

## Experimental protocols

In this study, we adapted six feature selection techniques (FST), two outlier removal techniques (ORT), and six cross-validation (CV) protocols: K2, K4, K5, K10, and JK-fold CV protocols, and ten different classifiers. We have performed two experimental protocols such as (i) to select best FST over CV protocols and ORT and (ii) comparison of the classifiers. Since the partitions K are random, we repeated the protocols with *T* = 10 trials in K2, K4, K5, and K10-folds CV protocols.

### Experiment 1: Select best cross-validation over outlier removal technique

The main objective of this section is to select the best CV protocols for both O1 and O2. The best CV protocols selection formula can be expressed as follows. Where, $\mathcal{A}$ (f, c, p) represents the mean accuracy of over different protocols when feature selection technique is “f”, classifier types is “c”, and data types is “p”, and total number of feature selection techniques, classifier types, and data types are F, C, and P, respectively.

18 $$ \mathcal{A}\left({\mathrm{k}}_{{\mathrm{o}}_{\mathrm{i}}}\right)=\frac{\sum_{\mathrm{f}=1}^{\mathrm{F}=6}{\sum}_{\mathrm{c}=1}^{\mathrm{C}=10}{\sum}_{\mathrm{p}=1}^{\mathrm{P}=768}\mathcal{A}\ \left(\mathrm{f},\mathrm{c},\mathrm{p}\right)}{\mathrm{F}\times \mathrm{C}\times \mathrm{P}},\mathrm{i}=1,2. $$

### Experiment 2: Best feature selection techniques over K-fold CV and ORT

The experiment presented in this section chooses the optimal FST over CV protocols and ORT’s on the basis of classification accuracy, where, $\mathcal{A}\left(\mathrm{k},\mathrm{c},\mathrm{p},\right)$ represents the accuracy of the classifer computed when protocol type is “k”, classifier type is “c”, patient number is “p”, and total number of protocols types, classifiers, and patients are: K, C, and P, then the mean accuracy of the performance of classification algorithms are evaluated in terms of measures.

19 $$ \mathcal{A}\left({\mathrm{f}}_{{\mathrm{o}}_{\mathrm{i}}}\right)=\frac{\sum_{\mathrm{k}=1}^{\mathrm{K}=5}{\sum}_{\mathrm{c}=1}^{\mathrm{C}=10}{\sum}_{\mathrm{p}=1}^{\mathrm{P}=768}\mathcal{A}\ \left(\mathrm{k},\mathrm{c},\mathrm{p}\right)}{\mathrm{K}\times \mathrm{C}\times \mathrm{P}},\mathrm{i}=1,2. $$

### Experiment 3: Comparison of the classifiers

The main objective of this experiment is to compare classification techniques based on classification accuracy and then select the best classifier. In this experiment, we adapted ten classifiers on both data: (i) data that contains outlier (O1) and (ii) impute outlier by the median (O2). For each dataset same FST and five sets of CV protocols are used. And compute the mean accuracy of all classifiers over protocols for both O1 and O2 datasets.

20 $$ \mathcal{A}\left({\mathrm{c}}_{{\mathrm{o}}_{\mathrm{i}}}\right)=\frac{\sum_{\mathrm{k}=1}^{\mathrm{K}=5}{\sum}_{\mathrm{f}=1}^{\mathrm{F}}{\sum}_{\mathrm{f}=1}^{\mathrm{F}=6}{\sum}_{\mathrm{p}}^{\mathrm{P}=768}\mathcal{A}\left(\mathrm{k},\mathrm{f},\mathrm{p}\right)}{\mathrm{K}\times \mathrm{F}\times \mathrm{P}},\mathrm{i}=1,2 $$

Where, $\mathcal{A}\left(\mathrm{k},\mathrm{f},\mathrm{p}\right)$ represents the accuracy of the classifer computed when protocol types is “k”, feature selection methods is “f”, and number of patients is “p”, and total number of protocols types, feature selection techniques, and number of patients are: K, F, and P. then the mean accuracy of the performance of classification algorithms are evaluated in terms of measures.

## Results

This section presents the results using the above two experimental protocol setup as discussed in section 4.1 (select best FST and protocols over and ORT) and section 4.2 (comparison of the classifiers). In the first experiment, best FST and CV protocols are estimated based on the criteria of the highest accuracy. The second experiment is to understand the behavior based the variation of the classification accuracy with respect to the different CV protocols. The results of these two experiments are shown in section 5.1 and section 5.2, respectively.

### Experiment 1: Select best feature selection techniques over K-fold CV and ORT

In this study, we adapted six FST as RF (F1), LR (F2), MI (F3), PCA (F4), ANOVA (F5), and FDR (F6) on both O1 and O2 datasets. For O1 and K2-protocol, F5-based feature selection technique gives the highest accuracy (81.94%). Increasing the value of K, ACC is also increased for both O1 and O2. On the contrary, F2 gives the highest ACC 84.66% of the same protocols for O2. In the same way, for K4, F4 and F2 give the highest ACC 82.73% and 86.16% for O1 and O2. For O2, RF gives the ACC (85.86%) for K10 and ACC (88.45%) for JK. There are also same results for O1. The details are given in Table 2. So we say that RF is the best FST for both O1 and O2.

**Table 2:** Comparison of mean accuracy of different protocols between O1 and O2 over FST

FST	O1					O2				
	K2	K4	K5	K10	JK	K2	K4	K5	K10	JK
F1	81.58	81.97	84.23	**84.66**	**86.05**	84.30	85.71	85.88	**85.86**	**88.45**
F2	81.84	81.45	83.23	83.56	86.77	84.66	86.16	84.40	84.40	88.40
F3	81.92	82.73	81.88	81.90	86.19	84.50	85.27	85.64	84.73	88.45
F4	81.48	81.98	83.09	82.23	85.66	83.71	84.60	83.73	84.60	87.91
F5	81.94	81.94	82.51	82.47	87.89	83.77	83.44	84.20	84.01	87.75
F6	71.48	73.51	74.90	74.82	78.13	75.53	75.82	76.77	77.35	79.32

Bold values indicate the highest classification accuracy

### Experiment 2: Comparison of the classifiers

For notational simplicity, we call the ten classifiers as: LDA (C1), QDA (C2), NB (C3), GPC (C4), SVM (C5), ANN (C6), Adaboost (C7), LR (C8), DT (C9), and RF (C10). This experiment is performed to investigate the comparison of performance of all classifiers with changing the K-folds CV protocols over ORT. Tables 3 and 4 show that increasing the value of K, classification accuracy is also increased for both O1 and O2 dataset. From these results, we intercept as (i) for K2 protocols, F1 and C10 classifier combination gives the highest accuracy (89.09% for O1 and 88.98 for O2) against the other classifiers because F1 extracts the most important features, (ii) increasing the value of K (2 to 4), the accuracy of C10 also increase. Tables 3 and 4 also show that F1 and C10 combination also gives the highest accuracy (89.79% for O1 and 89.58% for O2). Similarly it can be showed that for K10 protocols F1-C10 gives the accuracy 90.91% for and 92.26% for O2. JK protocols all feature selection based RF-based classifier combination gives 99.99~100.00% accuracy (both O1 and O2 datasets). So we say that F1 and C10 is the best combination for both O1 and O2 datasets.

**Table 3:** Comparisons of all classifiers and FST over protocols in terms of accuracy for O1

PT*	FST	C1	C2	C3	C4	C5	C6	C7	C8	C9	C10
K2	F1	77.21	73.83	76.56	85.23	85.18	78.88	86.33	78.54	86.93	**89.09**
	F2	77.76	76.38	77.86	84.71	84.92	76.88	85.76	79.17	87.24	87.73
	F3	77.24	74.27	77.03	83.88	83.93	81.98	87.16	78.57	86.25	88.88
	F4	77.55	75.13	77.45	82.89	82.99	79.82	85.08	79.90	86.51	87.47
	F5	77.73	75.36	77.55	84.48	85.08	78.70	85.78	79.56	87.21	87.97
	F6	69.64	67.97	68.78	72.29	71.61	68.62	73.67	71.20	75.18	75.81
K4	F1	76.30	73.49	75.73	86.25	86.46	79.90	86.41	78.39	86.93	**89.79**
	F2	77.34	74.84	76.93	85.68	83.39	75.68	84.74	79.27	88.02	88.65
	F3	78.02	75.26	77.71	85.52	84.90	81.56	87.55	80.10	86.93	89.79
	F4	77.55	75.10	77.86	84.11	82.97	80.62	85.21	80.94	86.98	88.49
	F5	78.96	76.72	78.28	85.68	86.35	80.10	85.00	80.73	87.66	89.06
	F6	70.94	68.85	69.95	75.83	73.70	70.52	75.57	73.28	77.92	78.49
K5	F1	80.32	77.40	79.48	88.51	87.21	81.17	87.34	82.40	88.57	**90.78**
	F2	79.22	77.27	78.64	87.53	86.56	79.68	86.30	80.78	87.34	88.96
	F3	77.47	73.77	76.62	84.81	84.22	81.36	85.91	78.96	87.01	88.70
	F4	77.92	76.30	78.18	85.39	84.03	82.66	86.36	82.60	87.86	89.55
	F5	77.79	75.19	77.21	85.58	84.81	81.04	86.30	80.32	87.73	89.16
	F6	71.62	71.82	71.88	78.70	75.39	72.53	74.55	74.74	78.57	79.22
K10	F1	77.62	74.48	77.03	85.57	85.00	80.93	86.63	79.62	87.07	89.59
	F2	78.18	76.36	78.44	88.05	85.58	78.31	88.70	81.69	89.35	**90.91**
	F3	76.75	73.38	76.10	84.81	83.12	81.69	85.71	80.39	86.88	90.13
	F4	77.92	75.32	77.92	85.84	83.25	78.96	85.45	82.34	86.23	89.09
	F5	76.75	75.58	77.53	86.88	85.19	81.43	84.29	80.91	87.01	89.09
	F6	72.73	69.87	71.56	79.09	75.58	72.34	74.29	75.97	77.66	79.09
JK	F1	77.92	76.05	77.44	89.01	90.41	82.16	99.92	78.32	89.28	**99.99**
	F2	78.27	81.22	78.78	88.12	89.24	83.20	99.49	79.16	90.23	**99.99**
	F3	78.09	76.24	77.04	88.49	89.99	83.82	99.87	78.37	90.03	**99.99**
	F4	77.77	75.60	77.34	86.77	88.97	82.98	99.62	80.29	87.31	**99.99**
	F5	83.67	83.84	82.84	88.42	88.28	79.06	98.63	84.13	88.59	**99.99**
	F6	70.10	70.20	68.88	77.30	76.63	75.82	96.02	71.26	76.97	**99.99**

Bold values indicate the highest classification accuracy

**Table 4:** Comparisons of accuracy of all classifiers and FST over protocols for O2

PT*	FST	C1	C2	C3	C4	C5	C6	C7	C8	C9	C10
K2	F1	83.88	83.78	84.37	87.34	86.15	79.14	86.67	85.29	86.54	**88.98**
	F2	84.40	84.11	84.71	86.82	85.05	77.66	84.87	85.23	86.02	87.76
	F3	83.39	83.41	83.70	86.48	85.21	79.87	85.42	84.82	85.05	87.66
	F4	82.50	82.84	82.50	85.16	84.32	80.03	84.19	83.70	85.13	86.72
	F5	83.10	83.83	83.10	85.81	83.93	77.63	84.11	84.27	84.82	86.75
	F6	76.56	76.28	76.90	77.37	75.99	72.16	72.16	77.94	73.88	76.04
K4	F1	85.31	85.21	85.05	88.28	86.61	79.58	87.50	87.45	87.03	**89.58**
	F2	83.80	84.17	84.06	87.24	85.99	79.90	86.35	85.83	86.41	88.96
	F3	85.10	84.43	84.90	88.28	86.61	79.79	86.09	86.61	85.83	89.48
	F4	83.49	83.75	83.23	86.41	84.90	79.90	84.84	85.42	85.89	88.13
	F5	82.45	82.50	82.40	86.15	83.91	77.71	83.33	84.01	85.26	86.67
	F6	76.61	75.99	76.82	78.33	75.47	72.03	71.15	79.06	76.46	76.30
K5	F1	84.94	84.48	84.29	88.70	86.49	79.29	86.49	86.82	87.53	**89.81**
	F2	82.47	82.27	82.27	86.75	84.94	77.99	86.88	85.26	86.17	88.96
	F3	84.22	84.61	83.77	88.31	86.82	79.94	87.08	85.91	86.10	89.61
	F4	82.08	82.08	81.95	86.17	83.70	80.58	83.05	84.35	85.71	87.66
	F5	83.12	83.38	83.38	87.08	84.35	79.16	82.21	84.94	85.91	88.44
	F6	77.08	76.36	77.14	78.57	77.34	72.66	73.57	79.68	76.62	78.64
K10	F1	83.38	84.16	82.73	89.35	86.49	81.17	84.42	85.97	87.27	**92.26**
	F2	85.45	85.71	85.97	89.61	86.62	78.83	87.27	88.05	87.79	90.26
	F3	82.86	82.60	83.38	88.05	85.19	76.75	86.62	86.62	86.49	88.70
	F4	82.60	83.77	82.73	87.14	83.64	79.74	82.86	87.40	87.79	88.31
	F5	82.86	83.12	82.73	87.79	85.06	76.49	82.73	86.36	85.97	87.01
	F6	77.66	77.27	77.53	80.13	77.14	75.32	72.99	80.26	76.62	78.57
JK	F1	84.12	84.31	83.74	88.43	88.66	80.40	99.82	84.78	90.20	**99.99**
	F2	84.01	84.79	84.03	88.72	88.30	79.14	99.24	85.66	90.14	**99.99**
	F3	84.12	84.31	83.74	88.44	88.62	80.50	99.82	84.78	90.20	**99.99**
	F4	83.45	84.00	82.76	87.30	87.45	81.67	99.81	84.13	88.58	**99.99**
	F5	81.10	82.26	83.66	88.17	89.09	83.21	99.82	81.39	90.23	**99.99**
	F6	71.10	70.32	69.88	78.30	77.63	77.82	97.02	72.26	76.97	**99.97**

Bold values indicate the highest classification accuracy

*Protocol Types

## Hypothesis validation and performance evaluation

### Hypothesis validation

As discussed in introduction section that the spirit of this study requires that when the missing values are replaced by the group median along with the replacement of the outliers by the median values, while using the random forest in ML framework should give the highest accuracy against the case when the outliers are either not removed or replaced by means. We demonstrate the results in Table 5, where we compared classification accuracy with outliers (O1) and without outliers (O2). We thus demonstrate that the hypothesis has been validated.

**Table 5:** Comparison of accuracy of classifier’s between O1 and O2 over protocols and FST

CT*	O1						O2					
	F1	F2	F3	F4	F5	F6	F1	F2	F3	F4	F5	F6
C1	78.14	78.15	77.51	77.74	78.22	71.23	84.03	83.73	83.68	82.82	83.04	76.98
C2	75.45	77.21	74.58	75.49	76.75	69.63	83.99	83.73	83.82	83.29	83.33	76.48
C3	77.56	78.13	76.90	77.75	78.7	70.54	83.77	83.67	83.73	82.63	82.89	77.10
C4	87.67	86.82	85.50	85.00	85.92	76.48	88.40	87.46	87.70	86.44	87.05	78.60
C5	87.44	85.94	85.23	84.44	85.85	74.07	86.72	85.97	86.37	84.80	85.11	76.48
C6	81.15	78.75	82.08	81.01	80.62	71.00	79.87	78.47	79.39	80.38	78.01	73.04
C7	89.92	89.00	89.24	88.34	88.39	74.52	88.38	89.07	89.06	86.95	86.20	72.47
C8	80.13	80.01	79.28	81.21	80.35	73.80	85.71	85.77	85.59	85.00	84.74	79.24
C9	88.16	88.44	87.42	86.98	87.88	77.33	87.24	87.11	86.85	86.62	86.14	75.90
C10	**91.35**	91.25	91.50	90.92	90.84	78.15	**92.29**	91.05	90.98	90.16	89.81	77.39

Bold values indicate the highest classification accuracy

* Classifier types

### Performance evaluation

#### Reliability

**Fig. 4:**
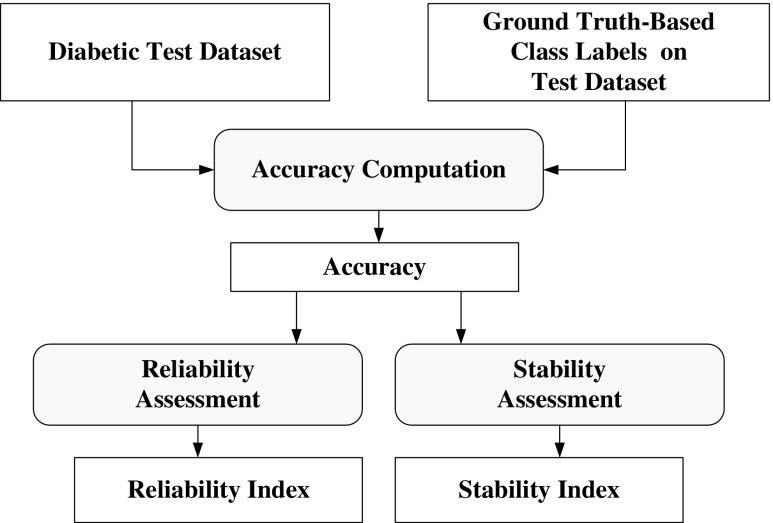
Performance evaluations of machine learning system

Reliability and stability index of the ML system is required for evaluation of the performance of the ML system. This can be seen in Fig. 4. The reliability index (RI) has been calculated by the ratio of the standard deviation of the classification accuracy and mean of the classification accuracy over data size (N). The system reliability index (ξ_N_) is calculated by the following formula as:

21 $$ {\upxi}_{\mathrm{N}}\left(\%\right)=\left(1-\frac{\upsigma_{\mathrm{N}}}{{\boldsymbol{\upmu}}_{\mathrm{N}}}\right)\times 100 $$

where, σ_N_ is the standard deviation and **μ**_N_ is the mean of all accuracies for FST and ORT’s. The system reliability index of $\overline{\upxi}$ by taking the mean of all data can be expressed as follows:

22 $$ \overline{\upxi}\left(\%\right)=\left(\frac{\sum_{\mathrm{n}=1}^{\mathrm{N}}{\upxi}_{\mathrm{n}}}{\mathrm{N}}\right) $$

**Fig. 5:**
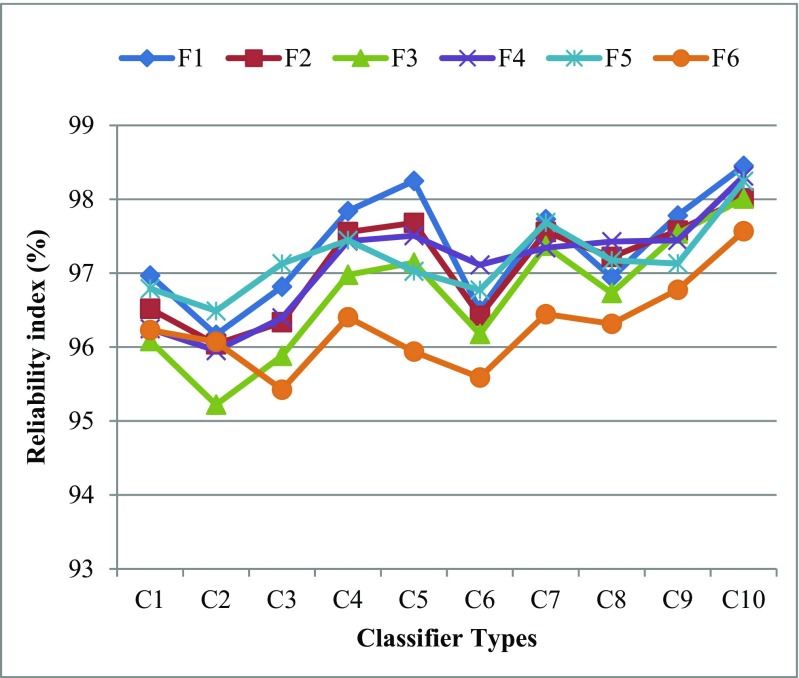
Comparison of all classifiers over different FST’s based on RI for O1

**Fig. 6:**
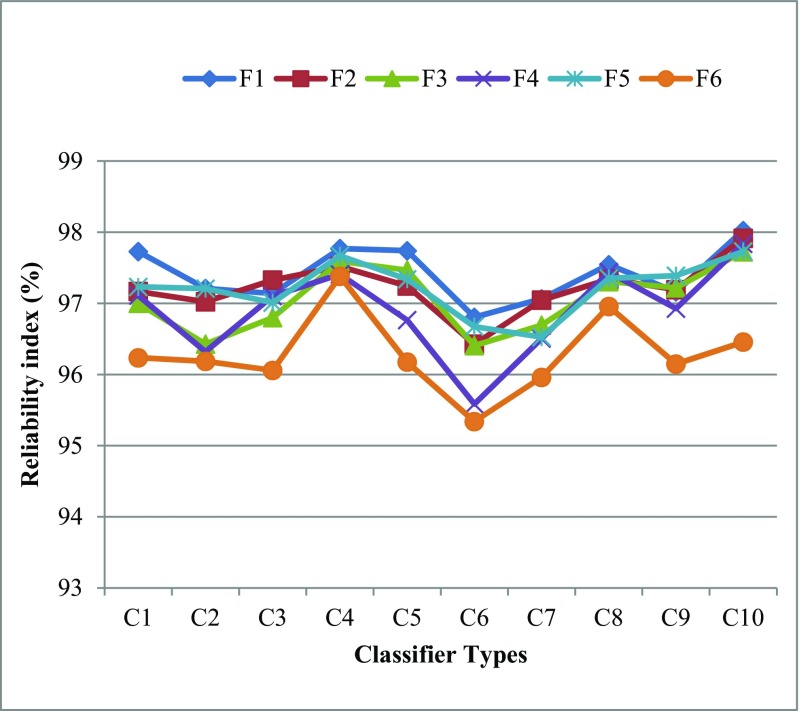
Comparison of all classifiers over different FST’s based on RI for O2

Figures 5 and 6 show that the reliability index (RI) for all F_i_-C_j_ (*i* = 1, 2…, 6 and j = 1, 2… 10) based 60 combinations as data size increases for O1 and O2 datasets. Further, the system reliability index has been computed by averaging the reliability indexes corresponding to all data sizes as shown Table 6 for O1 and Table 7 for O2 which confirms the best performance of F1 and C10 based combination for O1 and O2.

**Table 6:** Comparison of all classifiers over different FST’s based on RI for O1

FST	C1	C2	C3	C4	C5	C6	C7	C8	C9	C10
F1	96.97	96.17	96.82	97.84	98.25	96.50	97.73	96.94	97.78	**98.45**
F2	96.52	96.04	96.34	97.56	97.68	96.42	97.56	97.22	97.58	98.01
F3	96.08	95.22	95.88	96.98	97.14	96.18	97.37	96.73	97.53	98.01
F4	96.25	95.95	96.40	97.43	97.51	97.11	97.34	97.43	97.44	98.31
F5	96.79	96.49	97.13	97.44	97.03	96.77	97.68	97.17	97.13	98.24
F6	96.23	96.07	95.42	96.40	95.94	95.59	96.45	96.32	96.78	97.57

Bold values indicate the highest classification accuracy

**Table 7:** Comparison of all classifiers over different FST’s based on RI for O2

FST	C1	C2	C3	C4	C5	C6	C7	C8	C9	C10
F1	97.73	97.21	97.14	97.77	97.74	96.81	97.06	97.54	97.16	**98.02**
F2	97.17	97.02	97.33	97.52	97.24	96.43	97.05	97.34	97.19	97.91
F3	97.00	96.43	96.80	97.59	97.47	96.41	96.69	97.31	97.22	97.72
F4	97.11	96.32	97.10	97.41	96.77	95.58	96.50	97.44	96.93	97.84
F5	97.23	97.21	97.01	97.67	97.34	96.67	96.53	97.35	97.39	97.73
F6	96.24	96.18	96.06	97.38	96.18	95.34	95.96	96.96	96.15	96.46

Bold values indicate the highest classification accuracy

#### Stability analysis

Stability analysis defines the dynamics of control system. Here in our analysis data size can control the dynamics of overall system. We observed that at data system is stable within 2% tolerance limit.

## Discussion

This paper represents the risk stratification system to accurately classify diabetes disease into two classes namely: diabetic and control while input diabetic data contains outliers and replaced outliers by median. Moreover, sixty systems have been designed by cross combination of ten classifiers (LDA, QDA, NB, GPC, SVM, ANN, Adaboost, LR, DT, and RF) and six feature selection techniques (RF, LR,MI, PCA, ANOVA, and FDR) and their performances have been compared. The number of features has been selected with help of 0.90 cutoffs points for PCA while t-test has been adopted for LR, MI, FDR, respectively, and also F-test for ANOVA. The classification of diabetes disease has been implemented using one-against all approach for ten classifiers, i.e.*,* LDA, QDA, NB, GPC, SVM, ANN, Adaboost, LR, DT, and RF. Furthermore, four sets (K2, K4, K5, and K10) of cross-validation protocols has been applied for generalization of classification and this process has been repeated for T = 10 times to reduce the variability. For all sixty combinations, the experiments have been performed in one scenario as comparisons of outlier’s removal techniques varying different protocols. Performance evaluations of all classifiers are compared on the basis of ACC, SE, SP, PPV, NPV, and AUC in experiments with varying FST and CV protocols. The ML system was validated for stability and reliability.

The main focus of our study the following components: Comprehensive analysis of RF-based classifier against nine sets of classifiers: LDA, QDA, NB, GPC, SVM, ANN, Adaboost, LR, and DT, respectively while in input diabetic data, is replaced outliers by median and extract features. Our study shows that the classification must be improved if we replaced the missing values by group median and outliers by median and extract features by random forest and classification of diabetes disease by random forest. There are two reasons to improve the classification accuracy as (i) median missing values imputation while in existing papers, several authors were not using any missing imputation techniques and someone replaced missing values by mean; (ii) replaced outliers by median while in previous papers, authors did not use any methods to check outliers.

### Benchmarking different machine learning systems

**Fig. 7:**
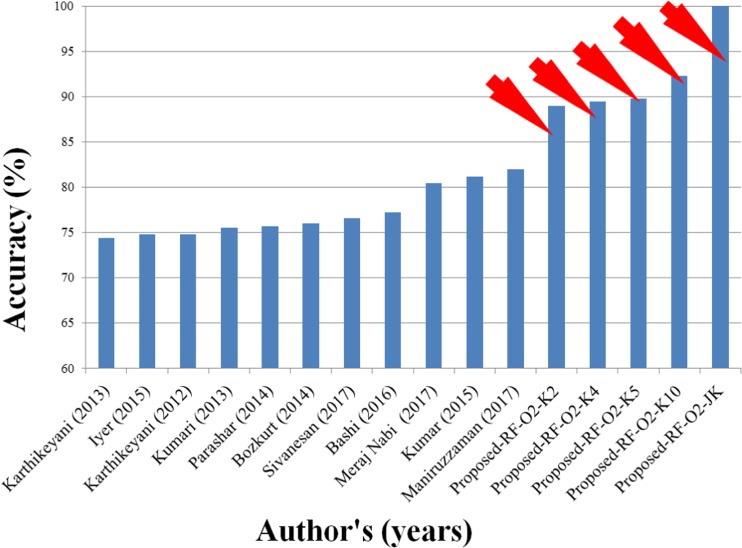
Comparison of our proposed method against the existing methods in literature. RED arrows shows the proposed work

There are several papers in literature on the diagnosis and classification of diabetic patients. Karthikeyani et al. [32] applied SVM with radial basis kernel on diabetes dataset. The dataset consisted of 8 attributes and 768 patients having 268 diabetes and 500 controls. They replaced these meaningless values with their mean and applied SVM to classify diabetes disease and demonstrated a classification accuracy of 74.80%. The same authors (Karthikeyani et al. [33]) extracted three features out of eight using partial least square (PLS) and applied LDA method to classify diabetes leading to an accuracy of 74.40%. Kumari and Chitra [34] introduced SVM with radial basis kernel function for classification. After deleting meaningless observations (zero contained observations), there were 460 observations. From those observations, 200 were used as training and rest of observations were used as a testing dataset, while the algorithm achieved a low accuracy of 75.50%. Parashar et al. [35] applied LDA to select the most importance features of diabetic disease and then selected two best features out of eight features. They also applied SVM and FFNN to classify diabetes disease and SVM gave the accuracy of 75.65%. Bozkurt et al. [36] introduced two ML techniques: AIS and ANN. ANN obtained higher accuracy of 76% compared to AIS. Iyer et al. [37] applied NB and DT for classification of diabetic patients. They replaced missing values with the mean and extracted two features out of eight using the correlation based feature selection (CFS) algorithm. They showed that DT obtained accuracy of 74.79%. Kumar Dewangan and Agrawal [38] used MLP and Bayes net classifiers, where MLP gave the highest accuracy of 81.19%. Bashir et al. [10] introduced Hierarchical Multi-level classifiers bagging with Multi-objective optimized Voting (HM-Bag Moov) technique to classify diabetes and compared to various classification techniques such as NB, SVM, LR, QDA, KNN, RF and ANN. They showed that HM-Bag Moov obtained an accuracy of 77.21%. Sivanesan et al. [39] proposed J48 algorithm to classify diabetic patients and obtained an accuracy of 76.58%. Meraj Nabi et al. [40] applied four different classifiers such as NB, LR, J48, RF, and obtained the best accuracy of 80.43% using LR. Recently, Suri’s team (Maniruzzaman et al. [9]) also applied four different classifiers such as LDA, QDA, NB, and GPC. They showed that GPC-based radial basis kernel gave the highest classification accuracy (~82%) with respect to others. From the above discussion, Table 8 and Fig. 7 confirm that our proposed F1 and C10 method is to identify the better diagnosis with an accuracy of 92.26% for K10 and nearly 100% for JK protocols compared to others. So our proposed system can be used to cross check of diagnosis of diabetes with the doctor’s assessment.

**Table 8:** Comparative performance of our proposed method against previous studies

SN	Authors	Year	Data size & class	# Features	MVIM^a^	ORT^b^	FST^c^	# selected Features	Classifier types	Performances measure (%)
1	Karthikeyani et al. [32]	2012	768 Controls: 500 Diabetic: 268	8	Mean	NA	NA	–	**SVM**	ACC:74.80
2	Karthikeyani et al. [33]	2013	768 Controls: 500 Diabetic: 268	8	NA	NA	PLS	3	**LDA**	ACC:74.40
3	Kumari and Chitra [34]	2013	460 Controls: 299 Diabetic:161	8	NA	NA	NA	–	**SVM**	ACC:75.50
4	Parashar et al. [35]	2014	768 Controls: 500 Diabetic: 268	8	NA	NA	LDA	2	**SVM,** FFNN	ACC:75.65
5	Bozkurt et al. [36]	2014	768 Controls: 500 Diabetic: 268	8	NA	NA	NA	–	AIS, **ANN**	ACC:76.00
6	Iyer et al. [37]	2015	768 Controls: 500 Diabetic: 268	8	Mean		CFS	4	**DT,** NB	ACC:74.79
7	Kumar Dewangan and Agrawal [38]	2015	768 Controls: 500 Diabetic: 268	8	NA	NA	None	–	**MLP**, Bayes Net	ACC:81.19
8	Bashir et al. [10]	2016	768 Controls: 500 Diabetic: 268	8	NA	NA	NA	–	NB, SVM, LR, QDA, KNN, RF, ANN, **HM-Bag Moov**	ACC:77.21 SE:77.65 SP:91.60 F-M:85.05
9	Sivanesan et al. [39]	2017	768 Controls: 500 Diabetic: 268	8	NA	NA	NA	–	J48	ACC:76.58
10	Meraj Nabi et al. [40]	2017	768 Controls: 500 Diabetic: 268	**8**	NA	NA	NA	–	NB, **LR**, J48, RF	ACC:80.43 MAE:0.30 MSE:0.37
11	Maniruzzaman et al. [9]	2017	768 Controls: 500 Diabetic: 268	8	Median	NA	NA	–	LDA, QDA, NB, **GPC**	ACC: 81.97 SE: 91.79 SP: 63.33 PV: 84.91 NPV: 62.50
12	**Proposed Method**	–	768 Controls: 500 Diabetic: 268	8	Group Median	Median	**RF** LR MI PCA, ANOVA FDR	4	LDA, QDA, NB, ANN, GPC, SVM, Adaboost, LR,DT, and **RF**	ACC: 92.26 SE: 95.96 SP: 79.72 PPV: 91.14 NPV:91.20 AUC: 93.11

Bold values indicate the highest classification accuracy

^a^Missing value imputation method; ^b^Outliers removal techniques; ^c^Feature selection techniques

Random forest showed encouraging results and identified the most significant features and classify of diabetes disease. It works well on both nonlinear and high dimensional data. In previous study, ML-based DM research has focused on only classification and prediction of diabetic patients. Here, RF capabilities to detect the relevant pattern in the data produced very meaningful results that correlate well with the criteria for diabetes diagnosis and with known risk factors. When we replaced the missing values and outliers are replaced by group mean and mean, then the RF yields 89% classification accuracy. This RF classification accuracy increased by 3% when missing values and outliers are replaced by group median and median, respectively.

### Strengths, weakness and extensions

This paper represents the risk stratification system to accurately classify of diabetes disease while there are 768 pregnant patients having two class diabetes and controls. Our study shows that RF-based feature selection technique along with RF-based classifiers with median based outlier’s removal techniques gives a classification accuracy of 92.26% for K10 protocols and nearly 99.99~100% for JK protocols (see Fig. 7). Nevertheless, the presented system can still be improved. Further, preprocessing techniques may be used to replace meaningless values by mean or median and outliers by mean or median. There are many other techniques of feature extraction, feature selection, and classification, and performances of presented combinations of system may be compared the other systems.

## Conclusion

Diabetes Mellitus (DM) is a group metabolic diseases in which blood sugar levels are too high. Our hypothesis was that if missing values and outliers are removed by group median and median values, respectively and such a data when used in ML framework using RF-RF combination for feature selection and classification should yield higher accuracy. We demonstrated our hypothesis by showing a 3% improvement and reaching an accuracy of nearly 100% in JK-based cross-validation protocol. Comprehensive data analysis was conducted consisting of ten classifiers, six feature selection methods and five sets of protocols, two outlier’s removal techniques leading to six hundred (600) experiments. Through benchmarking was analyzed and clear improvement was demonstrated. It would be interesting in future to see classification of other kinds of medical data to be adapted in such a framework creating a cost-effective and time-saving option for both diabetic patients and doctors.

## Electronic supplementary material

ESM 1 (DOCX 53 kb)

ESM 2 (DOCX 54 kb)

ESM 3 (DOCX 53 kb)

ESM 4 (DOCX 53 kb)

ESM 5 (DOCX 53 kb)

ESM 6 (DOCX 35 kb)
