# Supplementary material for: Accurate Diabetes Risk Stratification Using Machine Learning: Role of Missing Value and Outliers
Source: J Med Syst. 2018 Apr 10;42(5):92. doi: 10.1007/s10916-018-0940-7 (PMC5893681; doi:10.1007/s10916-018-0940-7)
Supplement: Supplementary file 1 — (DOCX 53 kb) [file 10916_2018_940_MOESM1_ESM.docx]

**Appendix A1**

Comparison of the performance evaluation of all classification and feature selection techniques for O1 and O2 techniques over K2 protocols are mentioned in this appendix (See Table 9).

Table 9. Comparisons of all classifiers and FST between O1 and O2 for K2 protocol.

| K2 protocol | | | | | | | | | | | | | |
| --- | --- | --- | --- | --- | --- | --- | --- | --- | --- | --- | --- | --- | --- |
| CT* | FST | O1 | | | | | | O2 | | | | | |
|  |  | ACC  (%) | SE  (%) | SP  (%) | PPV  (%) | NPV  (%) | AUC  (%) | ACC  (%) | SE  (%) | SP  (%) | PPV  (%) | NPV  (%) | AUC  (%) |
| C1 | F1 | 77.21 | 88.74 | 56.37 | 80.62 | 74.74 | 84.50 | 82.60 | 88.22 | 72.58 | 82.49 | 77.17 | 89.33 |
|  | F2 | 77.76 | 88.80 | 56.97 | 77.46 | 75.00 | 85.21 | 84.40 | 87.69 | 78.34 | 88.03 | 81.60 | 89.16 |
|  | F3 | 77.24 | 87.54 | 58.65 | 76.56 | 71.17 | 85.06 | 83.39 | 89.28 | 72.40 | 88.14 | 74.81 | 89.86 |
|  | F4 | 77.55 | 86.59 | 59.65 | 82.35 | 70.54 | 84.99 | 82.50 | 86.43 | 75.08 | 85.93 | 70.25 | 88.95 |
|  | F5 | 77.73 | 86.56 | 61.16 | 83.14 | 69.11 | 85.40 | 83.10 | 88.28 | 73.60 | 86.27 | 75.97 | 89.00 |
|  | F6 | 69.64 | 82.64 | 46.15 | 68.83 | 78.33 | 74.55 | 76.56 | 87.82 | 56.33 | 81.02 | 73.64 | 83.62 |
| C2 | F1 | 73.83 | 87.62 | 48.97 | 75.74 | 68.97 | 82.77 | 82.55 | 86.17 | 76.15 | 86.76 | 75.42 | 88.42 |
|  | F2 | 76.38 | 87.57 | 55.28 | 78.79 | 70.14 | 83.30 | 84.11 | 85.62 | 81.41 | 89.62 | 75.06 | 88.73 |
|  | F3 | 74.27 | 87.25 | 50.97 | 76.42 | 68.86 | 82.56 | 83.41 | 87.42 | 75.95 | 87.25 | 76.37 | 89.33 |
|  | F4 | 75.13 | 85.42 | 54.78 | 78.89 | 65.57 | 82.20 | 82.84 | 85.43 | 77.98 | 88.16 | 73.57 | 87.98 |
|  | F5 | 75.36 | 86.00 | 55.39 | 78.40 | 67.78 | 82.70 | 83.83 | 87.45 | 77.13 | 87.63 | 76.94 | 88.45 |
|  | F6 | 67.97 | 80.46 | 45.26 | 72.99 | 56.20 | 73.40 | 76.28 | 86.23 | 58.37 | 78.91 | 70.19 | 82.08 |
| C3 | F1 | 76.56 | 86.80 | 58.04 | 79.00 | 71.02 | 84.77 | 83.18 | 85.96 | 78.24 | 87.74 | 75.58 | 89.41 |
|  | F2 | 77.86 | 88.59 | 57.53 | 79.85 | 72.61 | 85.39 | 84.71 | 86.01 | 82.38 | 90.15 | 75.82 | 89.47 |
|  | F3 | 77.03 | 85.92 | 60.91 | 80.02 | 70.40 | 84.49 | 83.70 | 86.94 | 77.72 | 88.02 | 76.10 | 90.05 |
|  | F4 | 77.45 | 85.15 | 62.19 | 81.69 | 67.96 | 84.14 | 82.50 | 84.96 | 77.90 | 88.05 | 72.94 | 88.88 |
|  | F5 | 77.55 | 85.44 | 62.69 | 81.19 | 69.63 | 84.90 | 83.83 | 87.45 | 77.13 | 87.63 | 76.94 | 88.45 |
|  | F6 | 68.78 | 80.37 | 47.64 | 73.84 | 57.14 | 73.25 | 76.90 | 87.13 | 58.49 | 79.13 | 71.64 | 82.99 |
| C4 | F1 | 85.23 | 88.35 | 79.50 | 88.78 | 78.98 | 89.84 | 86.33 | 91.01 | 77.71 | 88.29 | 83.16 | 91.25 |
|  | F2 | 84.71 | 87.79 | 78.65 | 88.77 | 77.53 | 89.63 | 86.82 | 92.86 | 75.31 | 87.72 | 85.52 | 91.90 |
|  | F3 | 83.88 | 85.73 | 80.23 | 89.10 | 75.65 | 89.11 | 86.48 | 90.36 | 79.19 | 89.21 | 81.70 | 91.75 |
|  | F4 | 82.89 | 85.75 | 77.12 | 88.21 | 73.39 | 88.10 | 85.16 | 88.92 | 77.77 | 88.59 | 78.71 | 90.35 |
|  | F5 | 84.48 | 87.39 | 78.99 | 88.78 | 77.07 | 90.09 | 85.81 | 91.77 | 74.90 | 87.16 | 83.22 | 91.01 |
|  | F6 | 72.29 | 85.41 | 47.76 | 75.35 | 64.57 | 75.74 | 77.37 | 86.01 | 61.74 | 80.38 | 71.46 | 82.53 |
| C5 | F1 | 85.18 | 89.60 | 77.41 | 87.80 | 80.40 | 89.45 | 85.34 | 90.52 | 75.98 | 87.25 | 81.66 | 90.53 |
|  | F2 | 84.92 | 89.18 | 77.35 | 88.19 | 79.17 | 89.09 | 85.05 | 89.01 | 77.74 | 88.23 | 78.96 | 90.82 |
|  | F3 | 83.93 | 89.42 | 74.08 | 86.22 | 79.44 | 89.28 | 85.21 | 89.91 | 76.27 | 87.77 | 80.15 | 90.53 |
|  | F4 | 82.99 | 85.93 | 77.20 | 88.23 | 73.53 | 88.22 | 84.32 | 88.13 | 77.09 | 88.10 | 77.20 | 89.21 |
|  | F5 | 85.08 | 88.52 | 78.66 | 88.70 | 78.64 | 90.15 | 83.93 | 89.56 | 73.61 | 86.29 | 79.23 | 88.85 |
|  | F6 | 71.61 | 83.29 | 50.56 | 75.61 | 62.12 | 74.00 | 75.99 | 88.15 | 54.14 | 77.66 | 71.73 | 79.46 |

(Continued Table 9)

| CT* | FST | O1 | | | | | | O2 | | | | | |
| --- | --- | --- | --- | --- | --- | --- | --- | --- | --- | --- | --- | --- | --- |
|  |  | ACC  (%) | SE  (%) | SP  (%) | PPV  (%) | NPV  (%) | AUC  (%) | ACC  (%) | SE  (%) | SP  (%) | PPV  (%) | NPV  (%) | AUC  (%) |
| C6 | F1 | 78.88 | 84.07 | 69.80 | 83.50 | 70.97 | 80.39 | 78.72 | 87.69 | 62.56 | 80.97 | 73.79 | 80.41 |
|  | F2 | 76.88 | 84.24 | 63.32 | 81.29 | 68.01 | 80.26 | 77.66 | 83.78 | 66.29 | 82.36 | 68.53 | 77.50 |
|  | F3 | 81.98 | 86.49 | 73.97 | 85.77 | 75.28 | 85.81 | 79.87 | 87.50 | 65.55 | 82.74 | 73.78 | 79.65 |
|  | F4 | 79.82 | 80.24 | 78.99 | 88.33 | 66.90 | 85.01 | 80.03 | 84.57 | 71.37 | 85.01 | 70.64 | 84.51 |
|  | F5 | 78.70 | 83.62 | 69.53 | 83.80 | 69.53 | 82.76 | 77.63 | 84.32 | 65.46 | 81.87 | 69.35 | 78.16 |
|  | F6 | 68.62 | 87.33 | 34.25 | 71.10 | 60.24 | 67.03 | 72.16 | 89.52 | 40.67 | 73.36 | 70.11 | 70.88 |
| C7 | F1 | 86.33 | 89.36 | 80.88 | 89.48 | 80.68 | 93.30 | 85.10 | 89.30 | 77.51 | 87.82 | 80.01 | 91.55 |
|  | F2 | 85.76 | 90.06 | 77.59 | 88.42 | 80.57 | 91.67 | 84.87 | 87.47 | 80.04 | 89.20 | 77.16 | 92.34 |
|  | F3 | 87.16 | 89.55 | 82.97 | 90.51 | 81.33 | 93.88 | 85.42 | 89.27 | 78.11 | 88.55 | 79.57 | 92.25 |
|  | F4 | 85.08 | 86.24 | 82.75 | 90.84 | 75.22 | 92.67 | 84.19 | 87.33 | 78.20 | 88.49 | 76.38 | 91.38 |
|  | F5 | 85.78 | 88.53 | 80.71 | 89.63 | 78.93 | 92.90 | 84.11 | 86.78 | 79.14 | 88.49 | 76.57 | 91.03 |
|  | F6 | 73.67 | 77.05 | 67.71 | 81.50 | 61.82 | 80.67 | 72.16 | 76.07 | 65.19 | 79.87 | 60.14 | 78.05 |
| C8 | F1 | 78.54 | 88.12 | 60.71 | 80.78 | 74.82 | 84.82 | 84.38 | 86.70 | 80.12 | 88.88 | 77.01 | 89.35 |
|  | F2 | 79.17 | 88.22 | 61.77 | 81.51 | 74.06 | 85.40 | 85.23 | 87.75 | 80.48 | 89.47 | 77.95 | 89.31 |
|  | F3 | 78.57 | 88.05 | 60.95 | 80.67 | 74.12 | 85.11 | 84.82 | 87.19 | 80.34 | 89.34 | 77.06 | 89.88 |
|  | F4 | 79.90 | 84.57 | 70.45 | 85.17 | 70.32 | 85.27 | 83.70 | 86.81 | 77.54 | 88.32 | 75.95 | 88.90 |
|  | F5 | 79.56 | 85.89 | 67.72 | 83.63 | 72.60 | 85.73 | 84.27 | 87.49 | 78.08 | 88.29 | 77.66 | 89.01 |
|  | F6 | 71.20 | 83.20 | 49.10 | 75.14 | 61.72 | 74.60 | 77.94 | 89.71 | 56.54 | 79.03 | 75.69 | 83.59 |
| C9 | F1 | 86.93 | 92.37 | 77.16 | 88.07 | 85.04 | 90.24 | 85.36 | 92.18 | 73.05 | 86.24 | 84.32 | 89.24 |
|  | F2 | 87.24 | 95.74 | 70.88 | 86.26 | 90.15 | 90.20 | 86.02 | 92.21 | 74.58 | 87.23 | 84.00 | 89.70 |
|  | F3 | 86.25 | 92.07 | 75.42 | 87.44 | 85.28 | 89.54 | 85.05 | 92.32 | 71.23 | 85.95 | 83.49 | 89.40 |
|  | F4 | 86.51 | 95.71 | 68.44 | 85.76 | 89.18 | 88.83 | 85.13 | 92.51 | 70.85 | 86.02 | 83.88 | 87.74 |
|  | F5 | 87.21 | 95.54 | 71.63 | 86.52 | 89.99 | 90.89 | 84.97 | 95.05 | 66.50 | 84.04 | 88.37 | 88.44 |
|  | F6 | 75.18 | 89.87 | 47.74 | 76.38 | 74.28 | 78.81 | 73.88 | 83.30 | 56.28 | 78.37 | 66.83 | 76.99 |
| C10 | F1 | **89.09** | 94.06 | 79.99 | 89.58 | 88.41 | 94.25 | **87.40** | 93.55 | 76.10 | 87.79 | 87.10 | 93.29 |
|  | F2 | 87.73 | 94.10 | 75.28 | 88.08 | 87.72 | 93.17 | 87.76 | 93.91 | 76.22 | 88.15 | 87.16 | 93.66 |
|  | F3 | 88.88 | 93.70 | 80.05 | 89.63 | 87.77 | 94.25 | 87.66 | 93.43 | 76.81 | 88.38 | 86.24 | 93.62 |
|  | F4 | 87.47 | 93.37 | 75.74 | 88.51 | 85.71 | 93.31 | 86.72 | 94.81 | 70.95 | 86.41 | 88.48 | 92.79 |
|  | F5 | 86.93 | 94.80 | 72.00 | 86.53 | 89.45 | 92.30 | 87.97 | 94.35 | 75.88 | 88.10 | 88.01 | 93.98 |
|  | F6 | 75.81 | 83.24 | 62.15 | 80.51 | 67.55 | 81.39 | 76.04 | 87.90 | 54.54 | 77.80 | 71.77 | 80.94 |

*Classifier Types

Figure 8. Comparisons of accuracy of all classifiers and FST for K2 protocol for O1.

Figure 8. Comparisons of accuracy of all classifiers and FST for K2 protocol for O2.
