# Supplementary material for: Accurate Diabetes Risk Stratification Using Machine Learning: Role of Missing Value and Outliers
Source: J Med Syst. 2018 Apr 10;42(5):92. doi: 10.1007/s10916-018-0940-7 (PMC5893681; doi:10.1007/s10916-018-0940-7)
Supplement: Supplementary file 2 — (DOCX 54 kb) [file 10916_2018_940_MOESM2_ESM.docx]

**Appendix A2**

Comparison of the performance evaluation of all classification and feature selection techniques for O1 and O2 techniques over K4 protocols are mentioned in this appendix (See Table 10).

Table 10. Comparisons of all classifiers and FST’s between O1 and O2 for K4 protocol.

| K4 protocols | | | | | | | | | | | | | |
| --- | --- | --- | --- | --- | --- | --- | --- | --- | --- | --- | --- | --- | --- |
| CT* | FST | O1 | | | | | | O2 | | | | | |
|  |  | ACC  (%) | SE  (%) | SP  (%) | PPV  (%) | NPV  (%) | AUC  (%) | ACC  (%) | SE  (%) | SP  (%) | PPV  (%) | NPV  (%) | AUC  (%) |
| C1 | F1 | 76.30 | 88.04 | 55.94 | 73.57 | 71.15 | 84.50 | 85.10 | 89.16 | 77.62 | 92.00 | 76.12 | 91.15 |
|  | F2 | 77.34 | 89.79 | 56.74 | 78.10 | 78.18 | 85.17 | 85.31 | 89.54 | 77.11 | 90.28 | 83.33 | 90.73 |
|  | F3 | 78.02 | 88.46 | 59.51 | 74.29 | 75.00 | 85.88 | 83.80 | 87.02 | 77.51 | 87.12 | 76.67 | 90.59 |
|  | F4 | 77.55 | 88.39 | 58.65 | 80.15 | 82.14 | 85.63 | 83.49 | 88.52 | 74.25 | 84.67 | 83.64 | 89.59 |
|  | F5 | 77.73 | 86.56 | 61.16 | 83.14 | 69.11 | 85.40 | 82.45 | 87.98 | 72.36 | 82.96 | 84.21 | 88.56 |
|  | F6 | 70.94 | 84.23 | 46.20 | 73.94 | 74.00 | 76.44 | 76.61 | 88.36 | 54.42 | 81.56 | 70.59 | 83.79 |
| C2 | F1 | 73.49 | 87.83 | 48.50 | 74.93 | 69.49 | 82.57 | 84.43 | 86.84 | 80.00 | 88.82 | 76.96 | 90.38 |
|  | F2 | 74.84 | 87.72 | 53.50 | 75.76 | 72.55 | 82.96 | 85.21 | 87.18 | 81.34 | 90.04 | 76.82 | 89.64 |
|  | F3 | 75.26 | 87.66 | 53.37 | 77.09 | 71.04 | 83.45 | 84.17 | 86.26 | 80.15 | 89.68 | 74.72 | 89.85 |
|  | F4 | 75.10 | 86.20 | 55.87 | 77.33 | 69.76 | 82.76 | 83.75 | 86.87 | 77.99 | 88.17 | 76.21 | 88.57 |
|  | F5 | 75.36 | 86.00 | 55.39 | 78.40 | 67.78 | 82.70 | 82.50 | 86.15 | 75.98 | 86.77 | 75.15 | 88.19 |
|  | F6 | 68.85 | 82.04 | 44.26 | 73.51 | 56.44 | 75.38 | 75.99 | 85.71 | 57.68 | 79.37 | 68.11 | 82.52 |
| C3 | F1 | 75.73 | 86.14 | 57.66 | 78.10 | 70.47 | 84.10 | 84.90 | 86.17 | 82.41 | 90.01 | 76.54 | 90.96 |
|  | F2 | 76.93 | 88.40 | 57.93 | 77.68 | 75.16 | 84.91 | 85.05 | 87.07 | 81.07 | 89.86 | 76.76 | 90.39 |
|  | F3 | 77.71 | 85.78 | 63.46 | 80.79 | 71.37 | 85.30 | 84.06 | 85.58 | 80.89 | 90.01 | 73.71 | 90.39 |
|  | F4 | 77.86 | 86.54 | 62.78 | 80.27 | 72.72 | 84.64 | 83.23 | 86.72 | 76.87 | 87.62 | 75.54 | 89.50 |
|  | F5 | 77.55 | 85.44 | 62.69 | 81.19 | 69.63 | 84.90 | 82.40 | 86.46 | 74.99 | 86.35 | 75.28 | 88.46 |
|  | F6 | 69.95 | 82.38 | 46.77 | 74.48 | 58.33 | 74.88 | 76.82 | 86.78 | 58.03 | 79.73 | 69.76 | 83.09 |
| C4 | F1 | 86.25 | 88.23 | 82.44 | 90.09 | 80.58 | 90.51 | 88.28 | 92.12 | 81.10 | 90.04 | 85.75 | 92.49 |
|  | F2 | 85.68 | 88.41 | 80.84 | 88.77 | 81.37 | 89.83 | 88.28 | 93.34 | 78.20 | 89.42 | 86.25 | 92.81 |
|  | F3 | 85.52 | 89.22 | 78.46 | 88.45 | 80.74 | 90.58 | 87.24 | 92.75 | 75.35 | 88.71 | 84.31 | 92.67 |
|  | F4 | 84.11 | 84.69 | 82.96 | 89.83 | 75.61 | 89.09 | 86.41 | 89.17 | 80.32 | 89.91 | 80.42 | 91.16 |
|  | F5 | 84.48 | 87.39 | 78.99 | 88.78 | 77.07 | 90.09 | 86.15 | 93.41 | 72.68 | 86.35 | 86.83 | 90.66 |
|  | F6 | 75.83 | 86.91 | 53.96 | 78.57 | 70.02 | 79.25 | 78.33 | 85.49 | 63.86 | 82.53 | 70.21 | 82.74 |
| C5 | F1 | 86.46 | 90.98 | 78.80 | 88.23 | 83.39 | 90.17 | 86.61 | 90.33 | 79.97 | 89.07 | 82.05 | 91.40 |
|  | F2 | 83.39 | 88.08 | 75.81 | 85.79 | 79.77 | 89.09 | 86.61 | 91.04 | 77.75 | 88.97 | 81.83 | 92.10 |
|  | F3 | 84.90 | 88.34 | 78.71 | 88.22 | 79.03 | 90.42 | 85.99 | 89.65 | 78.67 | 89.37 | 79.15 | 91.88 |
|  | F4 | 82.97 | 87.89 | 74.62 | 85.72 | 77.97 | 89.20 | 84.90 | 88.72 | 77.83 | 88.22 | 78.72 | 90.38 |
|  | F5 | 85.08 | 88.52 | 78.66 | 88.70 | 78.64 | 90.15 | 83.91 | 89.90 | 73.26 | 85.93 | 80.03 | 88.87 |
|  | F6 | 73.70 | 84.39 | 53.95 | 77.58 | 64.74 | 78.17 | 75.47 | 88.77 | 50.46 | 77.29 | 70.33 | 80.20 |

(Continued Table 10)

| CT* | FST | O1 | | | | | | O2 | | | | | |
| --- | --- | --- | --- | --- | --- | --- | --- | --- | --- | --- | --- | --- | --- |
|  |  | ACC  (%) | SE  (%) | SP  (%) | PPV  (%) | NPV  (%) | AUC  (%) | ACC  (%) | SE  (%) | SP  (%) | PPV  (%) | NPV  (%) | AUC  (%) |
| C6 | F1 | 79.90 | 84.80 | 71.46 | 83.84 | 72.98 | 83.79 | 79.79 | 85.65 | 69.29 | 83.47 | 72.58 | 83.39 |
|  | F2 | 75.68 | 83.42 | 63.15 | 78.88 | 70.43 | 81.40 | 79.58 | 85.23 | 68.57 | 84.03 | 70.43 | 81.53 |
|  | F3 | 81.56 | 84.67 | 75.73 | 86.39 | 73.67 | 86.78 | 79.90 | 85.55 | 68.96 | 84.54 | 70.32 | 83.39 |
|  | F4 | 80.62 | 83.61 | 75.61 | 85.70 | 72.70 | 86.01 | 79.90 | 84.54 | 71.16 | 84.67 | 71.26 | 85.29 |
|  | F5 | 78.70 | 83.62 | 69.53 | 83.80 | 69.53 | 82.76 | 77.71 | 83.72 | 66.76 | 82.11 | 69.07 | 81.44 |
|  | F6 | 70.52 | 88.49 | 35.39 | 72.74 | 67.18 | 68.20 | 72.03 | 89.56 | 37.56 | 73.81 | 69.74 | 69.08 |
| C7 | F1 | 86.41 | 88.67 | 82.54 | 89.90 | 80.60 | 93.49 | 86.09 | 88.36 | 81.94 | 89.88 | 79.44 | 92.21 |
|  | F2 | 84.74 | 88.62 | 78.26 | 87.10 | 80.80 | 92.14 | 87.50 | 91.25 | 79.84 | 89.93 | 82.86 | 93.73 |
|  | F3 | 87.55 | 89.05 | 84.93 | 91.31 | 81.27 | 94.02 | 86.35 | 88.00 | 82.97 | 91.21 | 77.47 | 92.60 |
|  | F4 | 85.21 | 88.05 | 80.60 | 88.75 | 79.47 | 92.92 | 84.84 | 88.14 | 78.46 | 88.51 | 78.00 | 91.02 |
|  | F5 | 85.78 | 88.53 | 80.71 | 89.63 | 78.93 | 92.90 | 83.33 | 85.70 | 79.32 | 88.31 | 75.72 | 91.22 |
|  | F6 | 75.57 | 81.05 | 65.25 | 81.63 | 64.48 | 82.45 | 71.15 | 75.34 | 63.36 | 79.73 | 57.55 | 77.87 |
| C8 | F1 | 78.39 | 86.40 | 63.72 | 81.51 | 73.98 | 84.74 | 86.61 | 88.93 | 81.91 | 90.27 | 80.54 | 91.21 |
|  | F2 | 79.27 | 86.42 | 67.66 | 81.92 | 76.26 | 85.25 | 87.45 | 89.32 | 83.18 | 91.60 | 80.62 | 90.78 |
|  | F3 | 80.10 | 86.86 | 67.66 | 83.36 | 75.37 | 86.09 | 85.83 | 89.25 | 78.33 | 89.50 | 78.95 | 90.56 |
|  | F4 | 80.94 | 85.71 | 72.19 | 84.88 | 75.02 | 85.98 | 85.42 | 90.07 | 76.22 | 87.95 | 80.83 | 89.56 |
|  | F5 | 79.56 | 85.89 | 67.72 | 83.63 | 72.60 | 85.73 | 84.01 | 88.63 | 75.66 | 86.94 | 78.56 | 88.51 |
|  | F6 | 73.28 | 82.97 | 53.90 | 77.65 | 63.38 | 76.59 | 79.06 | 88.62 | 60.26 | 81.28 | 74.29 | 83.83 |
| C9 | F1 | 86.93 | 92.73 | 76.39 | 87.66 | 86.86 | 90.98 | 85.83 | 92.95 | 72.57 | 86.61 | 86.56 | 89.83 |
|  | F2 | 88.02 | 96.74 | 73.72 | 85.92 | 93.33 | 91.93 | 87.03 | 92.76 | 75.73 | 88.26 | 85.28 | 91.47 |
|  | F3 | 86.93 | 90.88 | 79.60 | 89.26 | 83.44 | 91.87 | 86.41 | 94.30 | 69.77 | 86.63 | 87.43 | 90.37 |
|  | F4 | 86.98 | 95.10 | 72.60 | 85.96 | 90.30 | 90.57 | 85.89 | 96.18 | 66.22 | 84.45 | 91.05 | 88.75 |
|  | F5 | 87.21 | 95.54 | 71.63 | 86.52 | 89.99 | 90.89 | 85.26 | 94.81 | 67.15 | 84.45 | 89.42 | 89.63 |
|  | F6 | 77.92 | 87.33 | 58.46 | 81.02 | 74.31 | 81.26 | 76.46 | 86.14 | 57.70 | 80.06 | 70.14 | 79.64 |
| C10 | F1 | **89.79** | 93.59 | 83.04 | 90.74 | 88.44 | 94.30 | **89.48** | 95.12 | 79.00 | 89.31 | 90.47 | 94.48 |
|  | F2 | 88.65 | 94.20 | 79.56 | 88.51 | 89.42 | 94.04 | 89.58 | 95.67 | 77.40 | 89.37 | 90.96 | 94.38 |
|  | F3 | 89.79 | 94.80 | 80.68 | 89.96 | 90.02 | 94.87 | 88.96 | 93.94 | 77.82 | 90.11 | 87.82 | 94.27 |
|  | F4 | 88.49 | 93.57 | 79.28 | 89.09 | 88.71 | 93.70 | 88.12 | 94.42 | 76.16 | 88.28 | 88.14 | 93.27 |
|  | F5 | 87.97 | 94.35 | 75.88 | 88.10 | 88.01 | 93.98 | 86.67 | 96.60 | 68.55 | 84.82 | 92.08 | 92.26 |
|  | F6 | 78.49 | 83.79 | 66.65 | 83.65 | 69.92 | 84.23 | 76.30 | 89.43 | 50.47 | 77.94 | 72.50 | 80.85 |

*Classifier Types

Figure 9. Comparisons of accuracy of all classifiers and FST of K4 protocol for O1.

Figure 10. Comparisons of accuracy of all classifiers and FST of K4 protocol for O2.
