# Supplementary material for: Accurate Diabetes Risk Stratification Using Machine Learning: Role of Missing Value and Outliers
Source: J Med Syst. 2018 Apr 10;42(5):92. doi: 10.1007/s10916-018-0940-7 (PMC5893681; doi:10.1007/s10916-018-0940-7)
Supplement: Supplementary file 3 — (DOCX 53 kb) [file 10916_2018_940_MOESM3_ESM.docx]

**Appendix A3**

Comparison of the performance evaluation of all classification and feature selection techniques for O1 and O2 techniques over K5 protocols are mentioned in this appendix (See Table 11).

Table 11. Comparisons of all classifiers and FST between O1 and O2 for K5 protocol.

| K5 protocol | | | | | | | | | | | | | |
| --- | --- | --- | --- | --- | --- | --- | --- | --- | --- | --- | --- | --- | --- |
| CT* | FST | O1 | | | | | | O2 | | | | | |
|  |  | ACC  (%) | SE  (%) | SP  (%) | PPV  (%) | NPV  (%) | AUC  (%) | ACC  (%) | SE  (%) | SP  (%) | PPV  (%) | NPV  (%) | AUC  (%) |
| C1 | F1 | 80.32 | 90.79 | 61.35 | 78.38 | 74.42 | 87.29 | 84.94 | 88.25 | 78.69 | 89.36 | 73.33 | 90.57 |
|  | F2 | 79.22 | 89.57 | 59.20 | 80.00 | 85.29 | 85.85 | 82.47 | 88.25 | 72.56 | 79.80 | 85.45 | 89.38 |
|  | F3 | 77.47 | 87.23 | 59.74 | 76.53 | 62.50 | 83.73 | 84.22 | 89.01 | 75.79 | 88.42 | 77.97 | 89.55 |
|  | F4 | 77.92 | 88.61 | 58.04 | 82.24 | 72.34 | 87.35 | 82.08 | 87.23 | 72.68 | 92.23 | 64.71 | 89.59 |
|  | F5 | 77.79 | 87.81 | 59.83 | 77.19 | 75.00 | 84.88 | 83.12 | 88.29 | 72.69 | 84.11 | 82.98 | 89.31 |
|  | F6 | 71.62 | 86.53 | 45.34 | 84.43 | 53.12 | 78.16 | 77.08 | 88.14 | 56.88 | 78.57 | 71.43 | 85.03 |
| C2 | F1 | 77.40 | 89.68 | 55.32 | 78.66 | 74.24 | 85.19 | 84.48 | 86.00 | 81.64 | 90.19 | 74.99 | 90.09 |
|  | F2 | 77.27 | 87.89 | 56.76 | 79.55 | 71.12 | 83.33 | 82.27 | 85.55 | 76.65 | 86.52 | 75.03 | 87.75 |
|  | F3 | 73.77 | 86.10 | 51.26 | 76.43 | 66.81 | 81.12 | 84.61 | 87.38 | 79.68 | 88.57 | 77.64 | 88.82 |
|  | F4 | 76.30 | 87.29 | 55.65 | 78.77 | 70.06 | 84.42 | 82.08 | 85.46 | 75.83 | 87.18 | 72.79 | 88.50 |
|  | F5 | 75.19 | 85.42 | 56.88 | 78.23 | 68.13 | 81.30 | 83.38 | 86.68 | 76.79 | 88.39 | 74.10 | 88.82 |
|  | F6 | 71.82 | 85.62 | 47.10 | 74.44 | 64.5 | 78.35 | 76.36 | 86.6 | 57.78 | 78.78 | 70.22 | 83.23 |
| C3 | F1 | 79.48 | 88.96 | 62.22 | 81.26 | 75.17 | 87.33 | 84.29 | 85.74 | 81.68 | 90.16 | 74.63 | 90.56 |
|  | F2 | 78.64 | 88.29 | 59.96 | 80.84 | 73.26 | 85.49 | 82.27 | 86.02 | 75.79 | 86.11 | 75.68 | 89.12 |
|  | F3 | 76.62 | 85.33 | 60.82 | 79.98 | 69.31 | 83.12 | 83.77 | 86.27 | 79.30 | 88.16 | 76.15 | 89.64 |
|  | F4 | 78.18 | 86.83 | 62.02 | 81.14 | 71.33 | 86.63 | 81.95 | 86.05 | 74.58 | 86.60 | 73.46 | 89.45 |
|  | F5 | 77.21 | 86.20 | 61.08 | 80.02 | 70.79 | 83.76 | 83.38 | 87.25 | 75.60 | 87.93 | 74.61 | 89.06 |
|  | F6 | 71.88 | 84.35 | 49.57 | 75.04 | 63.68 | 77.5 | 77.14 | 86.97 | 59.34 | 79.42 | 71.51 | 83.94 |
| C4 | F1 | 88.51 | 89.82 | 85.63 | 92.32 | 82.48 | 92.63 | 88.70 | 92.63 | 80.99 | 90.64 | 85.66 | 92.30 |
|  | F2 | 87.53 | 91.07 | 80.64 | 90.07 | 82.74 | 91.65 | 86.75 | 91.78 | 77.74 | 87.91 | 84.56 | 91.80 |
|  | F3 | 84.81 | 87.69 | 79.02 | 88.80 | 78.34 | 89.07 | 88.31 | 93.41 | 78.81 | 89.10 | 87.08 | 91.71 |
|  | F4 | 85.39 | 90.01 | 76.36 | 88.14 | 80.96 | 90.51 | 86.17 | 90.50 | 77.35 | 88.76 | 80.77 | 91.12 |
|  | F5 | 85.58 | 93.03 | 71.44 | 85.93 | 86.31 | 89.98 | 87.08 | 92.86 | 74.91 | 88.43 | 84.46 | 91.38 |
|  | F6 | 78.7 | 85.2 | 64.57 | 82.42 | 73.21 | 81.62 | 78.57 | 88.31 | 60.31 | 80.44 | 74.69 | 83.69 |
| C5 | F1 | 87.21 | 91.31 | 80.19 | 89.32 | 82.95 | 92.21 | 86.49 | 89.83 | 80.13 | 89.91 | 80.27 | 91.68 |
|  | F2 | 86.56 | 90.11 | 79.54 | 89.61 | 80.86 | 91.38 | 84.94 | 90.38 | 75.69 | 86.60 | 81.59 | 90.81 |
|  | F3 | 84.22 | 87.31 | 78.70 | 88.23 | 77.06 | 89.04 | 86.82 | 91.00 | 79.28 | 88.72 | 82.96 | 90.39 |
|  | F4 | 84.03 | 89.28 | 74.65 | 86.84 | 78.67 | 90.45 | 83.70 | 89.32 | 73.51 | 86.52 | 78.05 | 90.51 |
|  | F5 | 84.81 | 88.12 | 78.87 | 88.34 | 78.42 | 89.87 | 84.35 | 89.49 | 74.07 | 87.53 | 78.10 | 89.15 |
|  | F6 | 75.39 | 87.93 | 53.16 | 76.99 | 70.72 | 79.72 | 77.34 | 89.44 | 55.41 | 78.39 | 74.09 | 82.02 |

(Continued Table 11)

| CT* | FST | O1 | | | | | | O2 | | | | | |
| --- | --- | --- | --- | --- | --- | --- | --- | --- | --- | --- | --- | --- | --- |
|  |  | ACC  (%) | SE  (%) | SP  (%) | PPV  (%) | NPV  (%) | AUC  (%) | ACC  (%) | SE  (%) | SP  (%) | PPV  (%) | NPV  (%) | AUC  (%) |
| C6 | F1 | 81.17 | 85.57 | 73.46 | 85.47 | 73.26 | 85.31 | 79.29 | 85.42 | 67.49 | 83.77 | 70.38 | 83.68 |
|  | F2 | 79.68 | 85.15 | 69.08 | 84.17 | 71.07 | 83.85 | 77.99 | 82.80 | 69.69 | 82.71 | 69.74 | 82.00 |
|  | F3 | 81.36 | 85.24 | 74.43 | 85.93 | 73.18 | 86.19 | 79.94 | 86.37 | 68.71 | 83.20 | 73.51 | 82.89 |
|  | F4 | 82.66 | 85.46 | 77.76 | 87.80 | 74.29 | 87.85 | 80.58 | 86.11 | 70.86 | 84.87 | 72.38 | 86.22 |
|  | F5 | 81.04 | 85.81 | 72.84 | 85.07 | 74.04 | 85.25 | 79.16 | 84.73 | 68.00 | 84.32 | 68.65 | 83.59 |
|  | F6 | 72.53 | 85.00 | 48.86 | 75.27 | 64.36 | 72.51 | 72.66 | 90.68 | 38.77 | 73.47 | 73.86 | 70.99 |
| C7 | F1 | 87.34 | 88.83 | 85.00 | 91.47 | 80.43 | 94.82 | 86.49 | 88.68 | 82.39 | 90.87 | 78.85 | 92.37 |
|  | F2 | 86.30 | 92.13 | 75.47 | 87.73 | 83.31 | 92.67 | 86.88 | 91.06 | 79.45 | 88.71 | 83.33 | 92.86 |
|  | F3 | 85.91 | 87.03 | 84.09 | 90.90 | 77.79 | 93.21 | 87.08 | 89.01 | 83.64 | 90.66 | 80.76 | 93.24 |
|  | F4 | 86.36 | 89.37 | 81.28 | 89.88 | 80.52 | 93.75 | 83.05 | 86.47 | 77.05 | 87.73 | 74.47 | 91.23 |
|  | F5 | 86.30 | 88.61 | 82.17 | 89.99 | 79.84 | 93.80 | 82.21 | 84.69 | 77.27 | 88.34 | 71.69 | 91.28 |
|  | F6 | 74.55 | 81.02 | 62.68 | 79.77 | 64.34 | 83.12 | 73.57 | 76.86 | 67.55 | 81.09 | 61.98 | 80.22 |
| C8 | F1 | 82.40 | 89.08 | 69.94 | 85.02 | 78.48 | 87.66 | 86.82 | 91.59 | 77.01 | 88.96 | 83.09 | 90.54 |
|  | F2 | 80.78 | 90.28 | 61.95 | 82.14 | 78.29 | 86.18 | 85.26 | 85.88 | 83.69 | 90.35 | 77.64 | 89.45 |
|  | F3 | 78.96 | 83.41 | 70.07 | 84.14 | 70.70 | 84.00 | 85.91 | 88.33 | 81.18 | 89.56 | 79.48 | 89.57 |
|  | F4 | 82.60 | 86.60 | 74.64 | 86.74 | 75.05 | 87.48 | 84.35 | 87.03 | 79.68 | 89.42 | 76.38 | 89.54 |
|  | F5 | 80.32 | 89.14 | 64.14 | 82.12 | 77.40 | 84.93 | 84.94 | 89.01 | 76.34 | 88.68 | 77.94 | 89.26 |
|  | F6 | 74.74 | 84.02 | 55.95 | 78.15 | 66.37 | 78.14 | 79.68 | 90.96 | 59.64 | 80.22 | 79.1 | 85.11 |
| C9 | F1 | 88.57 | 94.00 | 78.19 | 88.97 | 88.47 | 93.49 | 87.53 | 93.10 | 76.51 | 88.78 | 86.03 | 90.89 |
|  | F2 | 87.34 | 94.82 | 72.68 | 87.09 | 88.71 | 92.22 | 86.17 | 94.06 | 72.01 | 85.76 | 87.95 | 90.34 |
|  | F3 | 87.01 | 95.05 | 72.51 | 86.40 | 89.23 | 91.29 | 86.10 | 93.69 | 72.18 | 85.96 | 87.36 | 89.20 |
|  | F4 | 87.86 | 95.13 | 74.39 | 87.62 | 90.28 | 92.72 | 85.71 | 95.77 | 65.68 | 84.60 | 90.65 | 89.91 |
|  | F5 | 87.73 | 94.35 | 75.63 | 87.79 | 89.27 | 91.62 | 85.91 | 94.91 | 67.36 | 86.04 | 89.37 | 90.75 |
|  | F6 | 78.57 | 86.08 | 61.67 | 82.59 | 77.17 | 82.59 | 76.62 | 88.72 | 54.84 | 78.4 | 74.12 | 81.22 |
| C10 | F1 | **90.78** | 95.55 | 81.69 | 90.66 | 90.93 | 95.75 | **89.81** | 94.26 | 80.88 | 90.73 | 88.71 | 94.18 |
|  | F2 | 88.96 | 94.46 | 78.40 | 89.48 | 88.69 | 94.58 | 88.96 | 95.25 | 77.37 | 88.44 | 91.06 | 93.66 |
|  | F3 | 88.70 | 95.15 | 76.64 | 88.39 | 90.24 | 93.64 | 89.61 | 94.61 | 80.29 | 89.86 | 89.60 | 93.81 |
|  | F4 | 89.55 | 95.18 | 78.63 | 89.52 | 90.52 | 94.70 | 87.66 | 93.87 | 74.11 | 88.21 | 88.19 | 93.00 |
|  | F5 | 89.16 | 94.98 | 78.67 | 89.06 | 89.91 | 94.23 | 88.44 | 96.22 | 72.59 | 87.76 | 90.66 | 93.29 |
|  | F6 | 79.22 | 85.22 | 65.61 | 83.1 | 75.79 | 84.55 | 78.64 | 91.62 | 55.1 | 79.05 | 79.55 | 82.96 |

*Classifier Types

Figure 11. Comparisons of accuracy of all classifiers and FST of K5 protocol for O1.

Figure 12. Comparisons of accuracy of all classifiers and FST of K5 protocol for O2.
