# Supplementary material for: Accurate Diabetes Risk Stratification Using Machine Learning: Role of Missing Value and Outliers
Source: J Med Syst. 2018 Apr 10;42(5):92. doi: 10.1007/s10916-018-0940-7 (PMC5893681; doi:10.1007/s10916-018-0940-7)
Supplement: Supplementary file 4 — (DOCX 53 kb) [file 10916_2018_940_MOESM4_ESM.docx]

**Appendix A4**

Comparison of the performance evaluation of all classification and feature selection techniques for O1 and O2 techniques over K10 protocols are mentioned in this appendix (See Table 12).

Table 12. Comparisons of all classifiers and FST for K10 protocol between O1 and O2.

| K10 protocol | | | | | | | | | | | | | |
| --- | --- | --- | --- | --- | --- | --- | --- | --- | --- | --- | --- | --- | --- |
| CT* | FST | O1 | | | | | | O2 | | | | | |
|  |  | ACC  (%) | SE  (%) | SP  (%) | PPV  (%) | NPV  (%) | AUC  (%) | ACC  (%) | SE  (%) | SP  (%) | PPV  (%) | NPV  (%) | AUC  (%) |
| C1 | F1 | 78.96 | 88.94 | 60.93 | 79.63 | 82.61 | 86.09 | 83.38 | 87.75 | 74.05 | 88.14 | 66.67 | 88.60 |
|  | F2 | 78.18 | 89.43 | 59.76 | 84.75 | 55.56 | 86.67 | 82.47 | 88.25 | 72.56 | 79.80 | 85.45 | 89.38 |
|  | F3 | 76.75 | 84.62 | 61.01 | 78.95 | 65.00 | 83.77 | 82.86 | 87.93 | 73.72 | 78.00 | 74.07 | 89.04 |
|  | F4 | 77.92 | 88.83 | 58.29 | 80.00 | 64.71 | 86.17 | 82.60 | 89.34 | 70.29 | 79.63 | 78.26 | 89.39 |
|  | F5 | 76.75 | 87.23 | 56.60 | 79.63 | 73.91 | 84.44 | 82.86 | 90.66 | 71.60 | 87.27 | 95.45 | 90.38 |
|  | F6 | 72.73 | 86.78 | 46.66 | 76.36 | 63.64 | 76.79 | 77.66 | 88.65 | 56.23 | 88.68 | 66.67 | 83.58 |
| C2 | F1 | 76.49 | 88.89 | 54.51 | 78.41 | 72.08 | 84.97 | 84.16 | 86.72 | 78.89 | 89.49 | 73.87 | 88.46 |
|  | F2 | 76.36 | 86.85 | 59.52 | 77.88 | 72.74 | 84.82 | 82.27 | 85.55 | 76.65 | 86.52 | 75.03 | 87.75 |
|  | F3 | 73.38 | 83.18 | 54.30 | 77.71 | 62.29 | 81.98 | 82.60 | 85.43 | 77.34 | 87.07 | 75.62 | 88.41 |
|  | F4 | 75.32 | 87.05 | 53.60 | 78.21 | 68.99 | 83.33 | 83.77 | 89.37 | 72.97 | 85.57 | 79.57 | 89.06 |
|  | F5 | 75.58 | 87.18 | 53.22 | 78.24 | 69.09 | 82.90 | 83.12 | 89.10 | 74.84 | 84.65 | 81.10 | 90.34 |
|  | F6 | 69.87 | 83.43 | 44.81 | 74.06 | 58.81 | 75.58 | 77.27 | 87.15 | 57.98 | 80.49 | 68.92 | 81.75 |
| C3 | F1 | 78.57 | 87.69 | 62.43 | 81.29 | 72.81 | 86.39 | 82.73 | 84.66 | 78.78 | 89.28 | 71.08 | 88.09 |
|  | F2 | 78.44 | 88.73 | 61.32 | 79.18 | 75.99 | 86.82 | 82.27 | 86.02 | 75.79 | 86.11 | 75.68 | 89.12 |
|  | F3 | 76.10 | 82.82 | 62.39 | 80.92 | 65.26 | 83.75 | 83.38 | 86.14 | 78.20 | 87.66 | 76.53 | 89.03 |
|  | F4 | 77.92 | 87.20 | 61.00 | 80.98 | 71.71 | 85.05 | 82.73 | 88.06 | 72.65 | 85.33 | 77.25 | 89.39 |
|  | F5 | 77.53 | 86.95 | 59.26 | 80.46 | 70.55 | 85.10 | 82.73 | 88.93 | 73.85 | 84.20 | 80.54 | 90.40 |
|  | F6 | 71.56 | 83.56 | 49.14 | 75.63 | 61.42 | 75.45 | 77.53 | 87.75 | 57.76 | 80.53 | 69.68 | 82.41 |
| C4 | F1 | 89.35 | 91.11 | 85.29 | 92.68 | 84.81 | 92.47 | 89.35 | 94.39 | 78.51 | 90.40 | 87.72 | 91.40 |
|  | F2 | 88.05 | 91.69 | 80.38 | 89.68 | 87.51 | 92.58 | 86.75 | 91.78 | 77.74 | 87.91 | 84.56 | 91.80 |
|  | F3 | 84.81 | 88.18 | 76.47 | 88.32 | 77.96 | 88.90 | 88.05 | 90.97 | 82.63 | 90.35 | 84.24 | 90.91 |
|  | F4 | 85.84 | 89.74 | 77.45 | 89.06 | 81.54 | 89.07 | 87.14 | 91.96 | 76.44 | 88.41 | 86.53 | 90.70 |
|  | F5 | 86.88 | 90.30 | 80.23 | 89.90 | 81.40 | 90.61 | 87.79 | 96.13 | 74.90 | 86.08 | 92.11 | 91.61 |
|  | F6 | 79.09 | 88.73 | 60.05 | 81.31 | 77.09 | 80.63 | 80.13 | 87.30 | 65.03 | 83.72 | 74.78 | 82.13 |
| C5 | F1 | 87.92 | 91.80 | 80.81 | 89.83 | 84.37 | 92.00 | 86.49 | 90.14 | 78.65 | 89.90 | 79.36 | 90.25 |
|  | F2 | 85.58 | 88.67 | 80.73 | 88.33 | 80.74 | 91.34 | 84.94 | 90.38 | 75.69 | 86.60 | 81.59 | 90.81 |
|  | F3 | 83.12 | 86.49 | 75.69 | 87.34 | 74.62 | 89.43 | 85.19 | 89.77 | 76.97 | 87.41 | 81.60 | 89.86 |
|  | F4 | 83.25 | 87.33 | 75.58 | 87.10 | 75.96 | 88.63 | 83.64 | 89.24 | 72.13 | 85.17 | 79.85 | 89.22 |
|  | F5 | 85.19 | 89.27 | 77.21 | 88.20 | 79.58 | 89.60 | 85.06 | 92.46 | 73.72 | 84.76 | 85.49 | 89.15 |
|  | F6 | 75.58 | 84.83 | 58.75 | 79.48 | 67.24 | 79.21 | 77.14 | 89.25 | 53.49 | 79.26 | 70.56 | 81.10 |

(Continued Table 12)

| CT* | FST | O1 | | | | | | O2 | | | | | |
| --- | --- | --- | --- | --- | --- | --- | --- | --- | --- | --- | --- | --- | --- |
|  |  | ACC  (%) | SE  (%) | SP  (%) | PPV  (%) | NPV  (%) | AUC  (%) | ACC  (%) | SE  (%) | SP  (%) | PPV  (%) | NPV  (%) | AUC  (%) |
| C6 | F1 | 83.64 | 85.92 | 79.80 | 88.59 | 74.92 | 87.89 | 81.17 | 85.91 | 71.27 | 86.15 | 70.94 | 84.08 |
|  | F2 | 78.31 | 85.22 | 66.64 | 80.92 | 72.78 | 83.91 | 77.99 | 82.80 | 69.69 | 82.71 | 69.74 | 82.00 |
|  | F3 | 81.69 | 85.61 | 73.40 | 86.16 | 72.31 | 85.65 | 76.75 | 83.80 | 64.59 | 80.71 | 69.42 | 80.44 |
|  | F4 | 78.96 | 83.11 | 71.35 | 84.72 | 69.13 | 85.74 | 79.74 | 85.32 | 69.36 | 83.03 | 72.78 | 86.24 |
|  | F5 | 81.43 | 85.86 | 72.75 | 85.83 | 73.56 | 85.21 | 76.49 | 82.64 | 66.88 | 79.92 | 70.63 | 83.20 |
|  | F6 | 72.34 | 88.16 | 40.83 | 74.31 | 74.71 | 70.64 | 75.32 | 91.86 | 42.38 | 76.61 | 78.85 | 72.13 |
| C7 | F1 | 89.61 | 91.47 | 86.32 | 92.50 | 84.34 | 95.45 | 84.42 | 86.63 | 79.90 | 90.05 | 74.23 | 91.14 |
|  | F2 | 88.70 | 92.46 | 81.99 | 89.77 | 86.65 | 93.76 | 86.88 | 91.06 | 79.45 | 88.71 | 83.33 | 92.86 |
|  | F3 | 85.71 | 85.68 | 86.40 | 91.94 | 75.30 | 93.96 | 86.62 | 89.05 | 81.86 | 89.80 | 81.71 | 91.77 |
|  | F4 | 85.45 | 89.68 | 77.47 | 88.51 | 79.61 | 92.70 | 82.86 | 87.78 | 73.36 | 85.53 | 76.63 | 89.79 |
|  | F5 | 84.29 | 84.97 | 83.04 | 90.54 | 74.51 | 93.03 | 82.73 | 87.70 | 75.98 | 85.25 | 79.06 | 91.81 |
|  | F6 | 74.29 | 78.73 | 66.44 | 81.69 | 62.13 | 81.67 | 72.99 | 76.20 | 66.50 | 82.05 | 58.57 | 77.77 |
| C8 | F1 | 82.99 | 88.75 | 71.42 | 86.24 | 78.70 | 86.57 | 85.97 | 91.15 | 74.79 | 88.64 | 80.32 | 88.53 |
|  | F2 | 81.69 | 87.28 | 69.37 | 83.99 | 81.15 | 86.89 | 85.26 | 85.88 | 83.69 | 90.35 | 77.64 | 89.45 |
|  | F3 | 80.39 | 87.94 | 62.19 | 83.31 | 74.96 | 83.81 | 86.62 | 89.11 | 81.69 | 90.00 | 81.78 | 89.13 |
|  | F4 | 82.34 | 91.57 | 63.76 | 83.50 | 82.54 | 86.37 | 87.40 | 90.99 | 78.94 | 89.67 | 84.09 | 89.34 |
|  | F5 | 80.91 | 87.63 | 67.12 | 84.94 | 77.98 | 84.90 | 86.36 | 89.29 | 81.39 | 88.81 | 82.26 | 90.30 |
|  | F6 | 75.97 | 89.44 | 50.12 | 77.60 | 71.26 | 76.95 | 80.26 | 93.71 | 53.90 | 80.33 | 82.44 | 83.44 |
| C9 | F1 | 89.09 | 94.93 | 77.73 | 89.32 | 90.52 | 94.32 | 87.27 | 94.53 | 71.82 | 87.66 | 87.17 | 87.45 |
|  | F2 | 89.35 | 94.31 | 80.50 | 89.45 | 90.01 | 92.83 | 86.17 | 94.06 | 72.01 | 85.76 | 87.95 | 90.34 |
|  | F3 | 86.88 | 92.09 | 74.59 | 88.37 | 85.24 | 91.99 | 86.49 | 90.93 | 78.39 | 88.74 | 84.73 | 89.85 |
|  | F4 | 86.23 | 94.92 | 69.15 | 85.59 | 89.06 | 90.32 | 87.79 | 96.81 | 70.68 | 86.24 | 92.75 | 89.99 |
|  | F5 | 87.01 | 93.15 | 75.25 | 87.99 | 86.09 | 90.29 | 85.97 | 89.38 | 78.32 | 88.09 | 84.99 | 90.45 |
|  | F6 | 77.66 | 92.49 | 48.29 | 77.77 | 79.22 | 80.64 | 76.62 | 87.52 | 54.17 | 79.75 | 72.27 | 80.12 |
| C10 | F1 | **91.82** | 95.36 | 86.15 | 92.50 | 90.97 | 95.90 | **92.26** | 95.96 | 79.72 | 91.14 | 91.20 | 93.11 |
|  | F2 | 90.91 | 94.66 | 83.55 | 91.31 | 91.99 | 95.40 | 88.96 | 95.25 | 77.37 | 88.44 | 91.06 | 93.66 |
|  | F3 | 90.13 | 93.83 | 81.93 | 91.37 | 88.16 | 94.59 | 88.70 | 94.64 | 77.83 | 88.76 | 90.74 | 92.54 |
|  | F4 | 89.09 | 95.31 | 76.26 | 89.04 | 91.59 | 93.77 | 88.31 | 94.52 | 75.92 | 88.08 | 90.57 | 91.97 |
|  | F5 | 89.09 | 97.66 | 72.92 | 87.35 | 94.81 | 94.30 | 87.01 | 96.23 | 71.93 | 85.25 | 92.51 | 92.48 |
|  | F6 | 79.09 | 85.60 | 65.84 | 84.08 | 75.29 | 84.06 | 78.57 | 92.11 | 51.68 | 79.33 | 79.44 | 80.74 |

Figure 13. Comparisons of accuracy of all classifiers and FST of K10 protocol for O1.

Figure 14. Comparisons of accuracy of all classifiers and FST of K10 protocol for O2.
