# Supplementary material for: Accurate Diabetes Risk Stratification Using Machine Learning: Role of Missing Value and Outliers
Source: J Med Syst. 2018 Apr 10;42(5):92. doi: 10.1007/s10916-018-0940-7 (PMC5893681; doi:10.1007/s10916-018-0940-7)
Supplement: Supplementary file 5 — (DOCX 53 kb) [file 10916_2018_940_MOESM5_ESM.docx]

**Appendix A5**

Comparison of the performance evaluation of all classification and feature selection techniques for O1 and O2 techniques over JK protocols are mentioned in this appendix (See Table 13).

Table 13. Comparisons of all classifiers and FST for JK protocol between O1 and O2.

| JK protocol | | | | | | | | | | | | | |
| --- | --- | --- | --- | --- | --- | --- | --- | --- | --- | --- | --- | --- | --- |
| CT* | FST | O1 | | | | | | O2 | | | | | |
|  |  | ACC  (%) | SE  (%) | SP  (%) | PPV  (%) | NPV  (%) | AUC  (%) | ACC  (%) | SE  (%) | SP  (%) | PPV  (%) | NPV  (%) | AUC  (%) |
| C1 | F1 | 77.92 | 88.08 | 58.96 | 80.00 | 72.48 | 85.11 | 84.12 | 88.41 | 76.13 | 87.35 | 77.86 | 90.12 |
|  | F2 | 78.27 | 90.48 | 55.49 | 79.20 | 76.02 | 84.55 | 84.01 | 88.41 | 75.81 | 87.18 | 77.78 | 89.94 |
|  | F3 | 78.09 | 88.20 | 59.22 | 80.18 | 72.94 | 85.07 | 84.12 | 88.41 | 76.13 | 87.35 | 77.86 | 90.12 |
|  | F4 | 77.77 | 88.12 | 58.47 | 79.89 | 72.69 | 85.76 | 83.45 | 88.58 | 73.88 | 86.35 | 77.65 | 89.71 |
|  | F5 | 81.10 | 91.79 | 61.16 | 81.53 | 80.00 | 85.88 | 83.67 | 88.60 | 74.49 | 86.38 | 77.95 | 89.56 |
|  | F6 | 70.10 | 82.83 | 42.61 | 72.88 | 60.37 | 68.83 | 71.10 | 85.83 | 43.61 | 73.88 | 62.37 | 75.83 |
| C2 | F1 | 76.05 | 88.20 | 53.37 | 77.92 | 70.80 | 84.14 | 84.31 | 87.19 | 78.94 | 88.54 | 76.76 | 89.83 |
|  | F2 | 81.22 | 89.08 | 66.56 | 83.25 | 76.57 | 87.45 | 84.79 | 86.82 | 81.02 | 89.51 | 76.71 | 89.47 |
|  | F3 | 76.24 | 88.60 | 53.17 | 77.93 | 71.43 | 84.15 | 84.31 | 87.19 | 78.94 | 88.54 | 76.76 | 89.83 |
|  | F4 | 75.60 | 86.41 | 55.44 | 78.35 | 68.61 | 83.55 | 84.00 | 87.24 | 77.96 | 88.07 | 76.61 | 89.70 |
|  | F5 | 82.26 | 90.98 | 66.01 | 83.31 | 79.68 | 89.06 | 83.84 | 86.98 | 77.99 | 88.06 | 76.25 | 89.34 |
|  | F6 | 70.20 | 82.41 | 44.03 | 71.78 | 60.22 | 74.70 | 70.32 | 84.41 | 44.03 | 73.78 | 60.22 | 75.70 |
| C3 | F1 | 77.44 | 86.60 | 60.33 | 80.29 | 70.71 | 85.11 | 83.74 | 85.80 | 79.88 | 88.84 | 75.10 | 89.96 |
|  | F2 | 78.78 | 89.58 | 58.64 | 80.16 | 75.10 | 86.27 | 84.03 | 86.61 | 79.22 | 88.61 | 76.02 | 89.77 |
|  | F3 | 77.04 | 85.73 | 60.83 | 80.33 | 69.56 | 84.68 | 83.74 | 85.80 | 79.88 | 88.84 | 75.10 | 89.96 |
|  | F4 | 77.34 | 86.18 | 60.86 | 80.42 | 70.24 | 85.01 | 82.76 | 86.20 | 76.34 | 87.17 | 74.78 | 89.57 |
|  | F5 | 83.66 | 90.95 | 70.07 | 85.01 | 80.58 | 88.93 | 82.84 | 86.01 | 76.94 | 87.44 | 74.67 | 89.50 |
|  | F6 | 68.88 | 81.94 | 44.53 | 71.96 | 56.85 | 73.37 | 69.88 | 82.94 | 45.52 | 73.96 | 58.85 | 74.37 |
| C4 | F1 | 89.01 | 92.12 | 83.19 | 91.09 | 85.00 | 93.04 | 88.43 | 94.09 | 77.88 | 88.81 | 87.61 | 93.59 |
|  | F2 | 88.12 | 96.42 | 72.64 | 86.81 | 91.60 | 65.10 | 88.72 | 94.58 | 77.79 | 88.82 | 88.51 | 93.59 |
|  | F3 | 88.49 | 92.01 | 81.93 | 90.48 | 84.61 | 92.51 | 88.44 | 94.10 | 77.89 | 88.82 | 87.63 | 93.60 |
|  | F4 | 86.77 | 91.17 | 78.56 | 88.83 | 82.72 | 92.25 | 87.30 | 90.43 | 81.45 | 90.10 | 82.04 | 93.01 |
|  | F5 | 88.17 | 96.73 | 72.22 | 86.66 | 92.22 | 65.10 | 88.42 | 93.74 | 78.49 | 89.06 | 87.09 | 93.11 |
|  | F6 | 77.30 | 85.34 | 62.31 | 80.46 | 70.35 | 82.35 | 78.30 | 86.34 | 63.31 | 81.46 | 71.35 | 83.35 |
| C5 | F1 | 90.41 | 93.10 | 85.40 | 92.25 | 86.90 | 92.72 | 88.66 | 91.83 | 82.74 | 90.85 | 84.45 | 93.89 |
|  | F2 | 89.24 | 96.77 | 75.19 | 87.92 | 92.58 | 93.60 | 88.30 | 92.50 | 80.45 | 89.82 | 85.19 | 93.80 |
|  | F3 | 89.99 | 92.29 | 85.70 | 92.33 | 85.64 | 92.72 | 88.62 | 91.79 | 82.72 | 90.83 | 84.38 | 93.86 |
|  | F4 | 88.97 | 91.24 | 84.75 | 91.78 | 83.83 | 93.14 | 87.45 | 90.65 | 81.49 | 90.14 | 82.36 | 92.89 |
|  | F5 | 89.09 | 96.40 | 75.45 | 87.99 | 91.83 | 93.58 | 88.28 | 92.86 | 79.74 | 89.53 | 85.70 | 92.84 |
|  | F6 | 76.63 | 88.16 | 55.12 | 78.13 | 72.51 | 81.64 | 77.63 | 89.16 | 56.12 | 79.13 | 73.51 | 82.64 |

* Classifier Types

(Continued Table 12)

| CT* | FST | O1 | | | | | | O2 | | | | | |
| --- | --- | --- | --- | --- | --- | --- | --- | --- | --- | --- | --- | --- | --- |
|  |  | ACC  (%) | SE  (%) | SP  (%) | PPV  (%) | NPV  (%) | AUC  (%) | ACC  (%) | SE  (%) | SP  (%) | PPV  (%) | NPV  (%) | AUC  (%) |
| C6 | F1 | 82.16 | 87.01 | 73.12 | 85.79 | 75.11 | 85.53 | 80.40 | 86.23 | 69.54 | 84.08 | 73.02 | 84.87 |
|  | F2 | 83.20 | 100.00 | 51.87 | 79.49 | 100.00 | 87.76 | 79.14 | 83.92 | 70.22 | 84.02 | 70.07 | 84.53 |
|  | F3 | 83.82 | 88.22 | 75.63 | 87.10 | 77.49 | 88.36 | 80.50 | 86.36 | 69.55 | 84.10 | 73.23 | 85.08 |
|  | F4 | 82.98 | 86.19 | 76.99 | 87.48 | 74.94 | 88.47 | 81.67 | 86.88 | 71.95 | 85.25 | 74.63 | 88.14 |
|  | F5 | 83.21 | 100.00 | 51.89 | 79.50 | 99.99 | 87.49 | 79.06 | 83.85 | 70.12 | 83.96 | 69.95 | 84.80 |
|  | F6 | 75.82 | 85.64 | 39.08 | 84.83 | 55.54 | 70.28 | 77.82 | 89.64 | 39.08 | 82.83 | 53.54 | 69.28 |
| C7 | F1 | 99.92 | 99.88 | 99.98 | 99.99 | 99.78 | 99.99 | 99.82 | 99.74 | 99.95 | 99.97 | 99.53 | 99.99 |
|  | F2 | 99.49 | 99.51 | 99.46 | 99.71 | 99.09 | 99.99 | 99.24 | 99.33 | 99.07 | 99.50 | 98.76 | 99.98 |
|  | F3 | 99.87 | 99.82 | 99.98 | 99.99 | 99.66 | 100.00 | 99.82 | 99.74 | 99.95 | 99.97 | 99.52 | 99.99 |
|  | F4 | 99.62 | 99.43 | 99.97 | 99.98 | 98.96 | 99.99 | 99.81 | 99.75 | 99.93 | 99.96 | 99.53 | 99.99 |
|  | F5 | 99.82 | 99.73 | 99.97 | 99.98 | 99.51 | 99.99 | 98.63 | 98.17 | 99.47 | 99.71 | 96.70 | 99.96 |
|  | F6 | 96.02 | 95.96 | 94.13 | 97.45 | 93.49 | 98.75 | 97.02 | 96.96 | 97.13 | 98.45 | 94.49 | 99.75 |
| C8 | F1 | 78.32 | 88.51 | 59.30 | 80.23 | 73.45 | 85.44 | 84.78 | 87.74 | 79.26 | 88.76 | 77.61 | 90.10 |
|  | F2 | 79.16 | 85.31 | 67.67 | 83.12 | 71.18 | 84.54 | 85.66 | 86.20 | 84.65 | 91.29 | 76.68 | 89.90 |
|  | F3 | 78.37 | 88.62 | 59.24 | 80.22 | 73.62 | 85.44 | 84.78 | 87.74 | 79.26 | 88.76 | 77.61 | 90.10 |
|  | F4 | 80.29 | 83.79 | 73.76 | 85.63 | 70.92 | 86.07 | 84.13 | 84.69 | 83.09 | 90.34 | 74.42 | 89.72 |
|  | F5 | 81.39 | 92.11 | 61.39 | 81.65 | 80.67 | 85.89 | 84.13 | 85.54 | 81.49 | 89.62 | 75.16 | 89.55 |
|  | F6 | 71.26 | 81.70 | 51.77 | 75.57 | 60.06 | 73.85 | 72.26 | 82.70 | 52.77 | 76.57 | 62.06 | 75.85 |
| C9 | F1 | 89.28 | 95.81 | 77.09 | 88.64 | 90.80 | 96.54 | 90.20 | 92.79 | 85.37 | 92.22 | 86.42 | 95.13 |
|  | F2 | 90.23 | 98.20 | 75.38 | 88.15 | 95.73 | 96.25 | 90.14 | 92.66 | 85.42 | 92.24 | 86.25 | 95.16 |
|  | F3 | 90.03 | 97.00 | 77.03 | 88.74 | 93.22 | 96.84 | 90.20 | 92.79 | 85.37 | 92.22 | 86.42 | 95.13 |
|  | F4 | 87.31 | 96.00 | 71.09 | 86.11 | 90.50 | 94.16 | 88.58 | 93.49 | 79.44 | 89.47 | 86.77 | 94.43 |
|  | F5 | 90.23 | 98.20 | 75.38 | 88.15 | 95.73 | 96.25 | 88.59 | 93.48 | 79.45 | 89.47 | 86.76 | 93.99 |
|  | F6 | 76.97 | 85.89 | 60.32 | 80.16 | 69.66 | 84.07 | 76.97 | 85.89 | 60.32 | 80.16 | 69.66 | 84.07 |
| C10 | **F1** | **99.99** | **99.99** | **99.98** | **99.99** | **99.99** | **100.00** | **99.99** | **99.99** | **99.98** | **99.99** | **99.99** | **100.00** |
|  | **F2** | **99.99** | **100.00** | **99.98** | **99.99** | **99.99** | **100.00** | **99.99** | **99.99** | **99.98** | **99.99** | **99.99** | **100.00** |
|  | **F3** | **99.99** | **99.99** | **99.98** | **99.99** | **99.99** | **100.00** | **99.99** | **99.99** | **99.98** | **99.99** | **99.99** | **100.00** |
|  | **F4** | **99.99** | **99.99** | **99.98** | **99.99** | **99.99** | **100.00** | **99.99** | **99.99** | **99.98** | **99.99** | **99.99** | **100.00** |
|  | **F5** | **99.99** | **100.00** | **99.98** | **99.99** | **99.99** | **100.00** | **99.99** | **99.99** | **99.98** | **99.99** | **99.98** | **100.00** |
|  | **F6** | **99.97** | **99.98** | **99.96** | **99.98** | **99.96** | **99.99** | **99.97** | **99.98** | **99.96** | **99.98** | **99.96** | **99.99** |

* Classifier Types

Figure 15. Comparisons of accuracy of all classifiers and FST of JK protocol for O1.

Figure 16**.** Comparisons of accuracy of all classifiers and FST of JK protocol for O2.
