# Supplementary material for: Accurate Diabetes Risk Stratification Using Machine Learning: Role of Missing Value and Outliers
Source: J Med Syst. 2018 Apr 10;42(5):92. doi: 10.1007/s10916-018-0940-7 (PMC5893681; doi:10.1007/s10916-018-0940-7)
Supplement: Supplementary file 6 — (DOCX 35 kb) [file 10916_2018_940_MOESM6_ESM.docx]

**Appendix A6**

Table 14. List of symbols.

| SN | Sym. | Descriptions | SN | Sym. | Descriptions |
| --- | --- | --- | --- | --- | --- |
| 1 | **X** | Data matrix. | 24 | O1 | Data contains outliers. |
| 2 | N | Total number of patients. | 25 | O2 | Impute outliers by median. |
| 3 | J | Total number of classes. | 26 | F1 | Random forest. |
| 4 | P | Total number of attributes. | 27 | F2 | Logistic regression. |
| 5 | $\boldsymbol{\mu}$ | Overall mean vectors. | 28 | F3 | Mutual information. |
| 6 | **I** | Identity vectors of 1. | 29 | F4 | Principal component analysis. |
| 7 | **S** | Sample variance-covariance matrix. | 30 | F5 | One-way analysis of variance. |
| 8 | $\lambda_{j}$ | J^th^ eigenvalues. | 31 | F6 | Fisher discriminant analysis. |
| 9 | e_j_ | J^th^ eigenvectors. | 32 | K2 | 2-fold cross-validation. |
| 10 | R | Cutoff points of PCA. | 33 | K4 | 4-fold cross-validation. |
| 11 | m | Number of reduced features. | 34 | K5 | 5-fold cross-validation. |
| 12 | N_j_ | Number of j^th^ class patients. | 35 | K10 | 10-fold cross-validation. |
| 13 | $\mu_{j}$ | J^th^ sample mean vectors. | 36 | C1 | Linear discriminant analysis. |
| 14 | $\mathbf{S}_{j}^{2}$ | J^th^ sample variance. | 37 | C2 | Quadratic discriminant analysis. |
| 15 | p-value | Probability values | 38 | C3 | Naïve bayes. |
| 16 | $\mathbf{S}_{w}$ | Within scatter matrix. | 39 | C4 | Gaussian process classification. |
| 17 | $\mathbf{S}_{B}$ | Between scatter matrix. | 40 | C5 | Support vector machine. |
| 18 | P(X,Y) | Joint probability distribution of X and Y. | 41 | C6 | Artificial neural network. |
| 19 | P(X) | Marginal probability distribution of X. | 42 | C7 | Adaboost. |
| 20 | P(Y) | Marginal probability distribution of Y. | 43 | C8 | Logistic regression. |
| 21 | $\xi_{N}$ | Reliability index. | 44 | C9 | Decision tree. |
| 22 | $\sigma_{N}$ | Standard deviation of all accuracies. | 45 | C10 | Random forest. |
| 23 | $\boldsymbol{\mu}_{N}$ | Mean of all accuracies. |  |  |  |

Table 15. List of Abbreviations.

| SN | Abbre. | Full form | SN | Abbre. | Full form |
| --- | --- | --- | --- | --- | --- |
| 1 | DM | Diabetes mellitus. | 27 | JK | Jackknife protocols. |
| 2 | PID | Pima Indian diabetes. | 28 | TP | True positive. |
| 3 | UCI | University of California Irvine. | 29 | TN | True negative. |
| 4 | FST | Feature selection techniques. | 30 | FP | False positive. |
| 5 | RF | Random forest. | 31 | FN | False negative. |
| 6 | LR | Logistic regression. | 32 | ACC | Accuracy. |
| 7 | MI | Mutual information. | 33 | SE | Sensitivity. |
| 8 | PCA | Principal component analysis. | 34 | SP | Specificity. |
| 9 | ANOVA | One-way analysis of variance. | 35 | PPV | Positive predictive value. |
| 10 | FDR | Fisher discriminant analysis. | 36 | NPV | Negative predictive value. |
| 11 | LDA | Linear discriminant analysis. | 37 | ROC | Receiver operating curves. |
| 12 | QDA | Quadratic discriminant analysis. | 38 | AUC | Area under the curve. |
| 13 | NB | Naïve Bayes. | 39 | RI | Reliability index. |
| 14 | GPC | Gaussian process classification. | 40 | F-M | F-measure. |
| 15 | SVM | Support vector machine. | 41 | MAE | Mean absolute error. |
| 16 | ANN | Artificial Neural network. | 42 | RMSE | Root mean square error. |
| 17 | DT | Decision tree. | 43 | IQR | Inter-quartile range. |
| 18 | Adaboost | Adaptive boosting. | 44 | MLE | Maximum likelihood estimator. |
| 19 | PLS | Partial least square. | 45 | PIM | Permutation importance index. |
| 20 | SD | Standard deviation. | 46 | GIM | Gini importance index. |
| 21 | FFNN | Feed forward neural networks. | 47 | OOB | Out of bag. |
| 22 | BPA | Back-propagation. | 48 | ORT | Outlier removal techniques. |
| 23 | NA | Not applied. | 49 | KNN | K-nearest neighborhood. |
| 24 | MLP | Multilayer perceptron. | 50 | CFS | Correlation based feature selection. |
| 25 | AIS | Artificial immune systems. |  |  |  |
| 26 | HM  BagMoov | Hierarchical Multi-level classifiers bagging with Multi-objective optimized Voting. | | | |
